# Phosphorylation of pRb: mechanism for RB pathway inactivation in *MYCN*‐amplified retinoblastoma

**DOI:** 10.1002/cam4.1010

**Published:** 2017-02-17

**Authors:** Kathryn G. Ewens, Tricia R. Bhatti, Kimberly A. Moran, Jennifer Richards‐Yutz, Carol L. Shields, Ralph C. Eagle, Arupa Ganguly

**Affiliations:** ^1^ Department of GeneticsPerelman School of MedicineUniversity of PennsylvaniaPhiladelphiaPennsylvania; ^2^ Department of PathologyThe Children's Hospital of PhiladelphiaPhiladelphiaPennsylvania; ^3^ Department of PathologyThe Perelman School of Medicine at the University of PennsylvaniaPhiladelphiaPennsylvania; ^4^ Oncology ServicesWills Eye HospitalThomas Jefferson UniversityPhiladelphiaPennsylvania; ^5^ Department of PathologyWills Eye HospitalThomas Jefferson UniversityPhiladelphiaPennsylvania

**Keywords:** Childhood‐onset ocular tumor, *MYCN* amplification, pRb and ppRb expression in RB, pRb inactivation by phosphorylation, *RB1* mutation‐negative retinoblastoma

## Abstract

A small, but unique subgroup of retinoblastoma has been identified with no detectable mutation in the retinoblastoma gene (*RB1)* and with high levels of *MYCN* gene amplification. This manuscript investigated alternate pathways of inactivating pRb, the encoded protein in these tumors. We analyzed the mutation status of the *RB1* gene and *MYCN* copy number in a series of 245 unilateral retinoblastomas, and the phosphorylation status of pRb in a subset of five tumors using immunohistochemistry. There were 203 tumors with two mutations in *RB1* (*RB1* ^−/−^, 83%), 29 with one (*RB1* ^+/−^, 12%) and 13 with no detectable mutations (*RB1* ^+/+^, 5%). Eighteen tumors carried *MYCN* amplification between 29 and 110 copies: 12 had two (*RB1* ^−/−^) or one *RB1* (*RB1* ^+/−^) mutations, while six had no mutations (*RB1* ^+/+^). Immunohistochemical staining of tumor sections with antibodies against pRb and phosphorylated Rb (ppRb) displayed high levels of pRb and ppRb in both *RB1* ^+/+^ and *RB1* ^+/−^ tumors with *MYCN* amplification compared to no expression of these proteins in a classic *RB1* ^−/−^, *MYCN*‐low tumor. These results establish that high *MYCN* amplification can be present in retinoblastoma with or without coding sequence mutations in the *RB1* gene. The functional state of pRb is inferred to be inactive due to phosphorylation of pRb in the *MYCN‐*amplified retinoblastoma without coding sequence mutations. This makes inactivation of *RB1* by gene mutation or its protein product, pRb, by protein phosphorylation, a necessary condition for initiating retinoblastoma tumorigenesis, independent of *MYCN* amplification.

## Introduction

In 1971, Alfred Knudson studied a series of cases of retinoblastoma, a childhood‐onset ocular tumor, and proposed the “two‐hit‐model” 1. According to this model, retinoblastoma is caused by biallelic inactivation of a single gene, *retinoblastoma1 (RB1)*. These two “hits” or mutations originally defined the concept of “tumor suppressor” genes. In heritable cases of retinoblastoma, the first hit is an inherited germline mutation, whereas the second hit is a somatic mutation; in sporadic cases, both hits are somatic in origin in a single retinal cell which initiates tumorigenesis 1.

Recently, a small subset of retinoblastoma with no detectable mutation in *RB1,* and amplification of the *MYCN* gene was identified 2. It was hypothesized that *MYCN* amplification initiated retinoblastoma tumorigenesis in the presence of functional pRb protein 2.

It is known that pRb can be inactivated by various mechanisms including genetic mutations and phosphorylation 3. To define which of the identified mechanisms is present in *MYCN*‐amplified retinoblastoma, we first investigated *MYCN* copy number and the mutations present in *RB1* in a series of 245 cases of unilateral retinoblastoma. We compared and contrasted the clinical features of the two groups of tumors classified by their *MYCN* amplification status. To explore the possibility of pRb inactivation by phosphorylation as an alternate pathway, we used immunohistochemical staining to evaluate the expression of four proteins: SKP2, a target of *MYCN* amplification, p27, a substrate for SKP2 ubiquitination thereby inhibiting pRb phosphorylation, total pRb, and phosphorylated pRb (ppRb).

## Materials and Methods

### Retinoblastoma specimens

A total of 245 unilateral retinoblastomas that had undergone complete screening for coding sequence and promoter region mutations in the *RB1* gene were studied 4, 5, 6. Ninety‐four tumors were collected following enucleation at Wills Eye Hospital (CLS), Thomas Jefferson University, Philadelphia, PA. These specimens were submitted to the histopathology laboratory at Wills Eye Hospital (RCE) for routine processing, diagnosis, and descriptive analysis. Frozen or formalin‐fixed paraffin‐embedded samples were sent to the Genetics Diagnostic Laboratory (GDL), Perelman School of Medicine, University of Pennsylvania (AG), Philadelphia, PA for genetic testing. An additional 151 cases of unilateral retinoblastoma were submitted to the GDL for genetic testing by 62 different sites in the US, Canada, Thailand, and Chile. Tumors used in this study were collected between 1982 and 2014. A small subset of 41 tumors was included in a previous study 7 and is indicated by an asterisk in Table S1. Pathology reports were available for 111 of the retinoblastoma samples (Table S2).

The Institutional Review Board of the University of Pennsylvania approved this research in accordance with an assurance filed with and approved by the U.S. Department of Health and Human Services. Since all patients were under the age of 18 years, written informed consent for use of tissues and data for research was obtained from a parent or legal guardian of all patients prior to genetic testing.

### DNA isolation and screening of the RB1 gene

DNA was isolated from frozen and formalin‐fixed, paraffin‐embedded retinoblastoma specimens using Qiagen DNeasy Blood and Tissue kits following manufacturer's protocols (Valencia, CA).

Mutation analysis of all 27 coding exons of the *RB1* gene, plus promoter and flanking intronic regions was performed by Sanger sequencing as previously described 4, 8. Methylation status of the promoter region, estimation of exonic copy number, and loss of heterozygosity (LOH) were carried out as previously described 4, 8. Mutations were annotated based on Genbank accession L11910.1 and compared against the LOVD‐*RB1* database 9.

### *MYCN* copy number determination and amplicon size

*MYCN* copy number was determined using an Applied Biosystems Taqman copy number assay (Hs00824796_cn, Life Technologies) using qPCR of tumor DNA following manufacturer's protocols. To characterize genome‐wide chromosomal changes and to determine the size of the *MYCN* amplicons, 38 retinoblastoma samples were genotyped using CytoScan HD SNP arrays following the manufacturer's instruction (Affymetrix, Santa Clara, CA). Array specific CEL files were generated in GeneChip Command Console Software. The CytoScan HD array data were imported into Affymetrix Chromosome Analysis Suite 3.0 (ChAS) software for analysis using filters for marker count = 50 and amplicon size = 100KB.

### Immunohistochemistry

Routinely processed formalin‐fixed and paraffin‐embedded tumor sections were immumostained with antibodies against Skp2/p45 (SC‐7164, rabbit anti‐human polyclonal [H‐435], Santa Cruz Biotechnology Inc, Dallas, TX), p27^Kip1^ (M 7203, mouse anti‐human monoclonal, Dako, Carpinteria, CA), and pRb (4H1 mAB 9309, mouse anti‐human, Cell Signaling, Danvers, MA) according to manufacturer's protocols using an automated immunostainer (Bond Max with Leica Bond Refine polymer, Leica Microsystems, Buffalo Grove, IL) with E1 (Skp2/p45) and E2 (p27^Kip1^ and pRb) retrieval systems at dilutions of 1:100, 1:200 and 1:300, respectively, followed by incubation at room temperature for one hour. Antibody to ppRb (S608) detects endogenous levels of pRb only when phosphorylated at Serine 608. Immunolocalization for ppRb was performed using a rabbit anti‐human polyclonal antibody (92181, Cell Signaling, Danvers, MA) and a nonautomated protocol as previously described 10.

### Statistical analysis

Statistical tests were carried out using the Fisher's exact probability or chi‐square test for contingency table analysis. Wilcoxon Mann–Whitney U‐test or Kruskal–Wallis test was used to compare the ages of patients in the different mutation categories. All analyses were two‐tailed and were carried out using IBM SPSS Statistics, version 23, vassarstats (http://vassarstats.net/) or SISA (http://www.quantitativeskills.com/sisa/).

## Results

### RB1 gene mutations and MYCN copy number changes in 245 unilateral retinoblastomas

The *RB1* gene was scanned for the presence of mutations in the coding exons and in the gene promoter region, exonic copy number changes, and methylation status of the *RB1* promoter sequence (Table 1 and Table S1). None of the tumors carried germline mutations. There were 203 tumors with two mutations in *RB1* (*RB1* ^−/−^, 83%), 29 with one (*RB1* ^+/−^, 12%) and 13 with no detectable mutations (*RB1* ^+/+^, 5%). A total of 435 somatic mutations were detected in 203 *RB1* ^−/−^ and 29 *RB1* ^+/−^ tumors. These included 199 (46%) point mutations, 136 (31%) instances of LOH, 57(13%) with promoter methylation, 36 (8%) exon deletions or duplications, and 7 (2%) complex rearrangements. There was no significant difference in the distribution of the types of mutations among tumors carrying one or two mutations (*P* = 0.11, Table 1).

**Table 1:** *RB1* mutation status in *MYCN*‐amplified (*MYCN*‐amp) and *MYCN*‐low retinoblastomas

*RB1* mutation status	Total (frequency)			*MYCN*‐amp (frequency)	*MYCN*‐low (frequency)	*RB1* ^−/−^		*RB1* ^+/−^	
						*MYCN*‐amp	*MYCN*‐low	*MYCN*‐amp	*MYCN*‐low
				*N* = 18	*N* = 227				
*RB1*−/−	203 (83%)			8 (44%)	195 (86%)				
*RB1* ^+/−^	29 (12%)			4 (22%)	25 (11%)				
*RB1* ^+/+^	13 (5%)			6 (33%)	7 (3%)				
*P*‐value^a^				<0.001					
Categories of mutations in *RB1* ^+/−^ and RB1^−/−^ tumors	*N* = 435 mutations	*RB1* ^−/−^ *N* = 406 mutations	*RB1* ^+/−^ *N* = 29 mutations	*N* = 20	*N* = 415	*N* = 16	*N* = 390	*N* = 4	*N* = 25
Point mutations	199 (46%)	190 (47%)	9 (33%)	10 (50%)	189 (46%)	9 (50%)	181 (46%)	1 (25%)	8 (32%)
LOH	136 (31%)	125 (31%)	11 (41%)	5 (24%)	131 (32%)	4 (28%)	121 (31%)	1 (25%)	10 (40%)
Promoter methylation	57 (13%)	52 (13%)	5 (18%)	2 (10%)	55 (13%)	2 (11%)	51 (13%)	1 (25%)	4 (16%)
Exonic deletions or duplications	36 (8%)	34 (8%)	2 (7%)	1 (5%)	35 (8%)	1 (6%)	33 (9%)	0	2 (8%)
Complex rearrangements	7 (2%)	5 (2%)	2 (7%)	2 (10%)	5 (1%)	1 (6%)	4 (1%)	1 (25%)	1 (4%)
*P*‐value^a^		0.11		0.14		0.36		0.58	

LOH, loss of heterozygosity.

a *P*‐values comparing number of tumors in *RB1* ^+/−^ with *RB1* ^−/−^, and *MYCN*‐amp with *MYCN*‐low categories were determined by Fisher's exact test (vassarstats) or Fisher 5*2 exact test (SISA).

**Figure 1:**
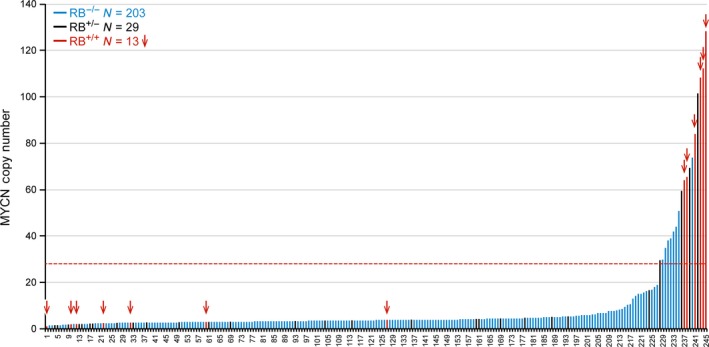
*MYCN* copy number in 245 retinoblastomas. Eighteen tumors carried *MYCN* copy number >29: 8 were *RB1* ^−/−^, 4 were *RB1* ^+/−^, and 6 were *RB1* ^+/+^. The dashed horizontal red line indicates a MYCN copy number of 29. (See Table S1 for UPEN‐RB‐IDs for samples labeled 1‐245 in the Figure).

We next estimated the copy number status of the *MYCN* oncogene in the 245 retinoblastomas, which ranged from 1 to 128 copies (Fig. 1). There were 227 (93%) tumors with less than 19 copies of *MYCN* (range 1–19), and 18 (7.3%) with copy number greater than 29 (range 30–128), eight of which were *RB1* ^−/−^ (Fig. 1 and Table S1). There were no tumors with *MYCN* copy number between 19 and 29.

### Characteristic features of MYCN‐amplified tumors

Of the 18 *MYCN*‐amplified tumors, eight (44%) were *RB1* ^−/−^, four (22%) were *RB1* ^+/−^, and six (33%) were *RB1* ^+/+^. Thus, twice as many tumors (66%) carried one or two *RB1* mutations compared to 33% of tumors with no mutations. This difference in the number of *MYCN*‐amplified retinoblastomas carrying two, one, or zero *RB1* mutations (eight, four, and six, respectively) compared to *MYCN*‐low tumors (195, 25, and seven, respectively) was significant (*P* < 0.001, Table 1). It can be seen in Table 1, however, that there was no significant difference in the distribution of the five categories of *RB1* mutations between the *MYCN*‐amplified and *MYCN*‐low tumors (*P* = 0.14), nor between *MYCN*‐amplified and *MYCN*‐low tumors within the *RB1* ^−/−^ (*P* = 0.36) or *RB1* ^+/−^ (*P* = 0.58) categories.

**Figure 2:**
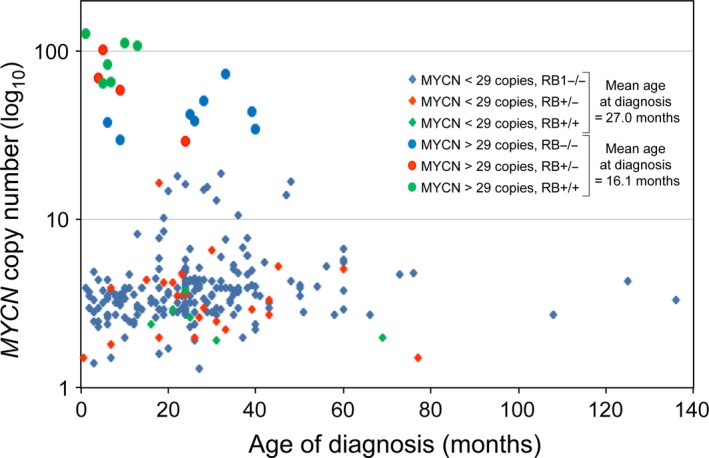
Age at diagnosis of 239 cases with retinoblastoma with and without *MYCN* amplification. Among the cases for whom age of onset was available, those with *MYCN* amplification developed retinoblastoma at a significantly younger age than those with *MYCN*‐low tumors (Mann–Whitney U‐test *P* = 0.012).

The age at which retinoblastoma was diagnosed was available for 239 cases (range = 0.5–136 months). There was no significant difference in the age of diagnosis and the presence of zero, one, or two *RB1* gene mutations (*P* = 0.33, Table S1, compiled data not shown). The median age at diagnosis for 13 RB1^+/+^ was 15.0 months (mean ± SD =18.7 ± 17.5, range = 1–69), 24 months (25.9 ± 17.4, 0.5–77) for 27 RB1^+/−^, and 24 months (26.7 ± 19.1, 1–136) for 199 RB1^−/−^ (*P* = 0.12). However, the 18 *MYCN*‐amplified tumors were diagnosed at a significantly earlier age: median of 9.5 months (16.1 ± 12.9, 1‐40) compared 24.0 months (27.0 ± , 19.0, 0.5‐136) for 221 *MYCN*‐low tumors (*P* = 0.012, Fig. 2).

**Figure 3:**
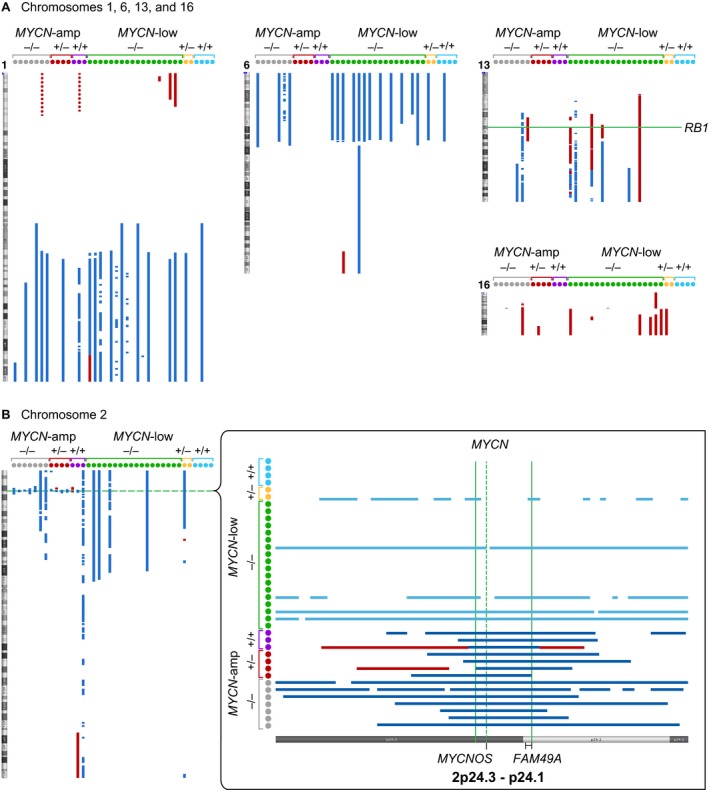
Copy number changes on chromosomes 1, 2, 6, 13, and 16 in 38 retinoblastomas. Copy number gains (vertical blue bars), or losses (vertical red bars) were visualized using Affymetrix ChAS software. *RB1* mutation status is indicated by −/−, +/‐, and +/+ notation separately in the *MYCN*‐amplified (‐amp) and *MYCN*‐low tumors. (A) Copy number changes on chromosomes 1, 6, 13, and 16 are indicated. The loss of the *RB1* gene region is marked by a horizontal green line. (B) Chromosome 2 with the *MYCN*‐amplified region is indicated by a horizontal dashed green line; blue bars indicate tumors with *MYCN* amplification. The figure on the right is an enlargement of the *MYCN* gene region, 2p24.3‐p24.1, with the darker blue lines denoting the region of *MYCN* amplification. The vertical dashed green line indicates the location of the *MYCN* gene, and the vertical solid green lines denote the minimum amplified region (chr2:15,891,962‐16,839,842). Among the *MYCN*‐low tumors, two had *MYCN* amplification of 3 which are indicated by lighter blue bars. The positions of two genes that map within the *MYCN* minimum amplified region, *MYCNOS* and *FAM49,* are marked.

Genome architecture, including whole genome chromosome copy number gains and losses, were determined for 38 retinoblastomas: 14 *MYCN*‐amplified and 24 *MYCN*‐low tumors. Classic retinoblastoma‐related chromosome copy number changes, including 1q gain, 6p gain, and 16q loss 2, 11, 12, 13, 14, 15, 16, were observed in 14 (37%), 15 (40%), and seven (18%) tumors (Fig. 3A), respectively; however, there was no significant difference in the fraction of tumors with these copy number changes between *MYCN*‐amplified and *MYCN*‐low retinoblastomas (Table 2). Figure 3B depicts copy number changes in the *MYCN* region on chromosome 2, showing the length of the *MYCN* amplicon in 14 tumors with *MYCN* amplification. The size ranged between 1 and 5 MB in 13 tumors, and was 11 MB in one tumor. The size of the minimum overlapping region that included the *MYCN* gene was 948KB and included the *MYCNOS* (noncoding anti‐sense RNA) gene and *FAM49A* exons 2‐11. Differences in copy number gains or losses for all other chromosomes were not significant.

**Table 2:** Chromosome and gene gains and losses in 38 retinoblastomas assessed using Affymetrix Cytoscan HD SNP arrays and analyzed with ChAS software

	Location^a^	Number with chromosome gain or loss (percent of total)	*MYCN*‐amplified (CN>29)	*MYCN*‐low	
		Total *N* = 38	*N* = 14	*N* = 24	P‐value^b^
Chromosome gain or loss					
1q gain		14 (37%)	4 (29%)	10 (42%)	0.50
6p gain		15 (40%)	3 (21%)	12 (50%)	0.10
16q loss		7 (18%)	1 (7.1%)	6 (25%)	0.23
Gene gain or loss					
*MDM4* gain^c^	Chr1:204,485,507‐204,527,248	16 (42%)	6 (43%)	10 (42%)	1.00
*OTX2* gain^c^	Chr14:57,267,425‐57,272,381	5 (13%)	2 (14%)	3 (12%)	1.00
*BCOR* loss^c^	ChrX:39,910,499‐39,956,719	3 (7.8%)	2 (14%)	1 (4.2%)	0.54
*KIF14* gain^d^	Chr1:200,520,625‐200,589,862	11 (29%)	3 (21%)	8 (33%)	0.49
*DEK* gain^d^	Chr6:18,224,400‐18,264,799	18 (47%)	4 (29%)	14 (58%)	0.10
*E2F3* gain^d^	Chr6:20,402,137‐20,493,945	17 (45%)	3 (21%)	14 (58%)	0.04
*CDH11* loss^d^	Chr16:64,980,683‐65,155,919	7 (18%)	1 (7.1%)	6 (25%)	0.23

a GRCh37/hg19 assembly.

b Fisher's exact, two‐tailed, P‐value (http://vassarstats.net/tab 2 × 2.html).

c McEvoy, et al.^7^

d Rushlow, et al.^2^

Copy number gains of *MDM4* and *OTX2,* and loss of *BCOR* in retinoblastoma have been reported previously 7, 12, 17. In our collection of retinoblastomas, *MDM4* and *OTX2* copy number gains were found in 16 (42%) and five (13%) of 38 retinoblastoma, respectively. Loss of *BCOR* was seen in three of 34 tumors (7.9%) (Table 2); all three carried two or one *RB1* mutations. No significant association was found between the presence of these copy number gains or losses, and *MYCN* amplification. In the previous study of *MYCN*‐amplified retinoblastoma, a lower frequency of copy number changes in four genes characteristic of retinoblastoma (*KIF14*,* DEK*,* E2F3*, and *CDH11*) was observed in *RB1* ^+/+^ *MYCN*‐amplified compared to *RB1* ^−/−^ tumors 2. We found no significant difference in the number of copies of *KIF14, DEK*, and *CDH11* between *MYCN*‐amplified and *MYCN*‐low tumors; however, there was a significant difference in copy number gains in *E2F3* where a smaller fraction of *MYCN*‐amplified tumors showed a gain (3/14, 21%) compared to *MYCN*‐low (14/24, 58%, *P* = 0.04) tumors (Table 2).

Pathology reports were available for 111 retinoblastomas and are summarized in Table S2: 12 were *MYCN* amplified and 99 were *MYCN* low. There was no significant association between the tumors carrying zero, one, or two *RB1* mutations and the presence of high‐risk features defined as invasion of the optic nerve to the level of the retrolamina, massive uveal/choroidal invasion and/or invasion of the anterior chamber, iris or ciliary body (*P* = 0.73) 18, 19. In addition, there was no significant difference between the degree of tumor differentiation and *MYCN* amplification status (*P* = 0.16) or tumors carrying zero, one, or two *RB1* mutations (*P* = 0.77).

Among the 12 *MYCN*‐amplified tumors with pathology reports, four (33%) had high‐ risk features compared to 23 of 99 *MYCN*‐low tumors (23%, *P* = 0.48). One of the four *MYCN*‐amplified, high‐risk tumors, (UPENN‐RB‐175) was *RB1* ^+/+^ and was diagnosed at 10 months, by which time the tumor had invaded both the retrolaminar portion of the optic nerve and showed massive choroidal invasion. However, overall, the age of diagnosis was not significantly different between the four patients whose tumors had high‐risk features and *MYCN* amplification and the 23 high‐risk, but *MYCN*‐low tumors (*P* = 0.16).

In terms of histological differentiation, 11 (92%) *MYCN*‐amplified tumors were poorly differentiated consisting of neuroblastic cells and few, if any rosettes; one tumor was described as mixed with respect to differentiation (Table S2 and S3). This distribution of the degree of differentiation was not significantly different from that found in *MYCN*‐low retinoblastoma: 63 (68%) undifferentiated/poorly differentiated, 21 (23%) well or moderately well differentiated with numerous Flexner–Wintersteiner rosettes, and seven (7.6%) with mixed status (*P* = 0.16, compiled data not shown).

### Immunohistochemical staining pattern

**Figure 4:**
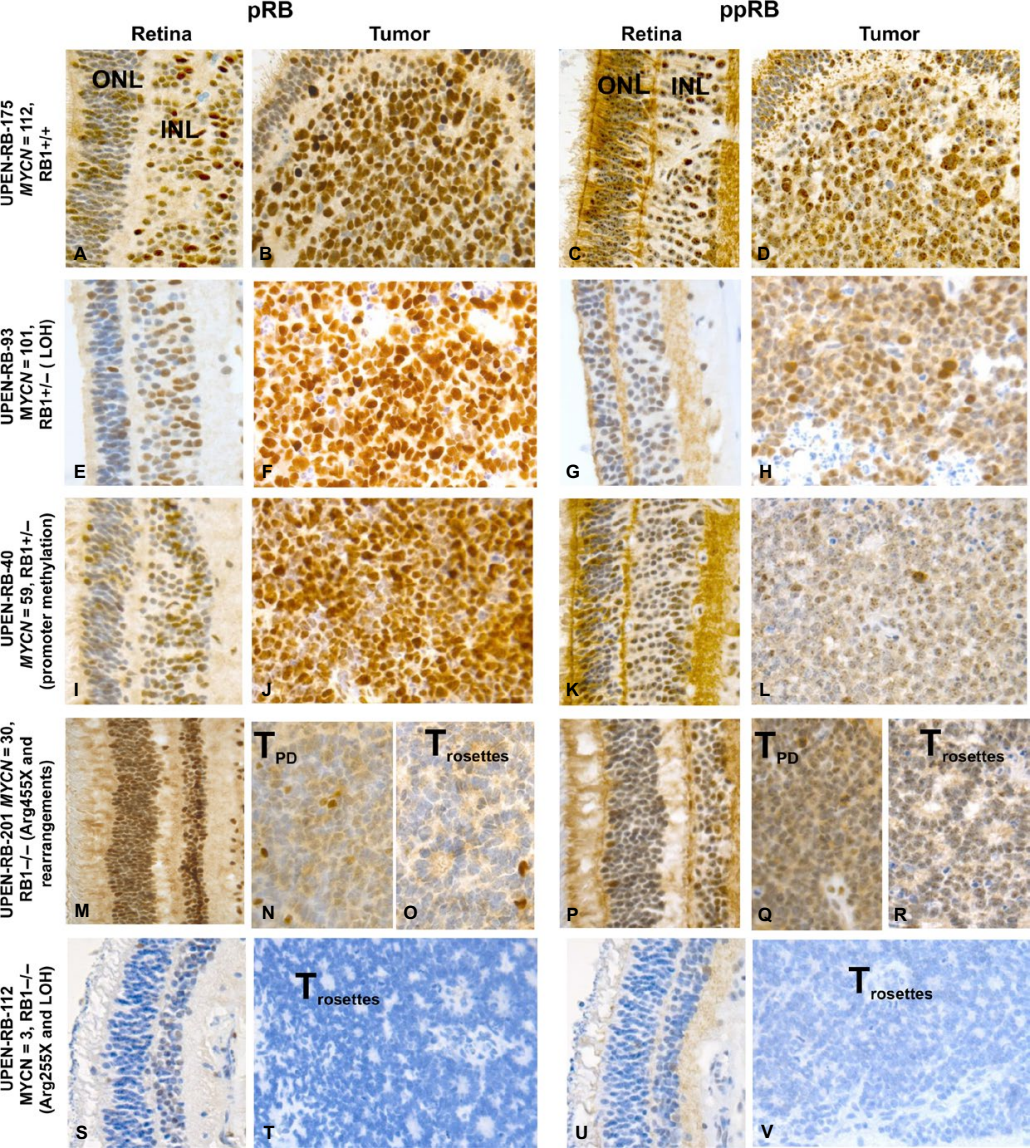
Expression of pRb and ppRb in the normal retina and in the tumor of four *MYCN*‐amplified and one *MYCN*‐low retinoblastoma identified by immunostaining with antibodies against the two proteins. The anti‐ppRb stain is specifically targeted against phosphorylated S608. In the retina images, the ONL is positioned on the left and the INL on the right. A moderate level of pRb and ppRb reactivity is seen in less than half of the cells in both the ONL and INL of MYCN‐amplified tumors UPEN‐RB‐175 (*RB1* ^+/+^, A, C) and the two *RB1* ^+/−^ tumors, UPEN‐RB‐93 (E, G) and UPEN‐RB‐40 (I, K). Most cells in both retinal layers were strongly positive for pRb (M), but not ppRb (P) in UPEN‐RB‐201 (*MYCN*‐amplified, but *RB1* ^+/−^). The INL of UPEN‐RB‐112 (*MYCN*‐low, *RB1* ^−/−^) shows weak reactivity for pRb in about 25% of cells and even fewer are positive for ppRb, the ONL is essentially negative for both proteins (S, U). The *MYCN*‐amplified tumors with zero (B) or one *RB1* mutation (F, J) are poorly differentiated with most cells strongly stained with pRb. Many cells are also positive with ppRb antibody (D, H, L) but the staining tends to be weaker. The regions of UPEN‐RN‐201 (*MYCN*‐amp and *RB1* ^−/−^) that are well differentiated with many FW rosettes show very little reactivity with pRb or ppRb (O, R), while scattered positive cells are present in the poorly differentiated regions (N, Q). Similarly, the well‐differentiated *RB1* ^−/−^, but *MYCN*‐low tumor, UPEN‐RB‐112, was essentially negative for pRb and ppRb (T, V). Abbreviations: inner (INL) and outer (ONL) nuclear layer of the retina; T_Rosettes_, region of tumor containing numerous rosettes; T_PD_, poorly differentiated region of the tumor. (Original magnification ×40).

We compared the relative levels of expression of SKP2, p27, pRb, and ppRb proteins in four *MYCN*‐amplified and one *MYCN*‐low retinoblastomas by immunohistochemistry (IHC) staining of tumor sections with antibodies specific for these proteins (Table 3 and Fig. 4, Figure S1 and Table S4).

**Table 3:** Tumor characteristics of five retinoblastoma immumostained with antibodies against pRb and ppRb

UPEN‐RB ID	MYCN Copy Number	*RB1* Mutations	Histopathological Features^a^	High Risk Features	Immunohistochemical Staining Patterns					
					Tumor		Normal Retina			
					pRb	ppRb	ONL^b^	INL^c^	ONL^b^	INL^c^
							pRb		ppRb	
UPEN‐RB‐175	112	*RB1* ^+/+^	PD‐few HW rosettes; large cells with nucleoli, 50% necrotic	Invasion of optic nerve and choroid	Most cells strongly positive	Most cells moderately to strongly positive	About 50% of cells moderately positive	About 25% strongly positive cells	Very few positive cells	30‐40% moderately positive cells
UPEN‐RB‐93	101	*RB1* ^+/−^	PD‐no rosettes; cells with prominent nucleoli	None	Most cells strongly positive	Most cells positive about 30% strongly stained	About 25% strongly positive cells	About 50% moderately positive cells	30‐40% moderately positive cells	About 50% weakly positive cells
UPEN‐RB‐40	59	*RB1* ^+/−^	PD‐no rosettes; extensively necrotic	None	Most cells strongly positive	Most cells are weakly staining but with a very few strongly stained	<25% strongly positive cells	About 50% moderately positive cells	Few moderately positive cells	30‐40% moderately positive cells
UPEN‐RB‐201	30	*RB1* ^−/−^	Mixed tumor differentiation; many FW rosettes	None	Rosettes: mostly negative but with a few strongly staining cells; PD areas: scattered positive cells	Rosettes: mostly negative; PD areas: scattered positive cells	>90% strongly positive	>90% strongly positive	Very few positive cells	>50% moderately positive cells
UPEN‐RB‐112	3	*RB1* ^*−/−*^	WD with many FW rosettes; extensively necrotic	None	Negative	Negative	Negative	About 25% weakly positive cells	Few weakly staining cells	Negative

a Histopathological feature: WD, well‐differentiated tumor; PD, poorly differentiated tumor; FW, Flexner–Wintersteiner rosettes; HW, Homer–Wright rosettes.

b ONL, outer layer of retina.

c INL, inner layer of retina.

Staining with anti‐SKP2 antibody was essentially negative in all five retinoblastomas, although a few scattered positive cells were present in the four *MYCN*‐amplified tumors (Fig. S1, panels A,C,E,G,H) and Table S4). The p27 staining pattern in UPEN‐RB‐175 was faint and diffuse (Fig. S1 B; *RB1* ^+/+^), while in UPEN‐RB‐93 and UPEN‐RB‐40 (Fig. S1, D,F; both *RB1* ^+/−^), it was patchy and focally strong, especially in UPEN‐RB‐40. Over 90% of tumor cells in UPEN‐RB‐201 (*RB1* ^−/−^) were strongly positive (Fig. S1, I,J). UPEN‐RB‐112 *(MYCN‐low, RB1* ^−/−^) was negative for SKP2 (Fig. S1, K), but showed very weak reactivity for p27 in some areas (Fig. S1, L) (Table S4).

The histopathological features, as well as pRb and ppRb (Ser608) IHC staining pattern in five retinoblastoma tumor sections and matched normal retina (defined as retinal tissue not in proximity to the tumor) are visualized in Figure 4 and described in Table 3. About 25–50% of cells in the inner (INL) and outer (ONL) nuclear layers of the normal retina were moderately to strongly stained with both pRb and ppRb antibodies in the *MYCN*‐amplified tumors UPEN‐RB‐175 (Fig. 4, panels A,C), UPEN‐RB‐93 (Fig. 4E,G), and UPEN‐RB‐40 (Fig. 4I,K) carrying zero or one *RB1* mutations. This would indicate that the pRb protein that is expressed is phosphorylated. Over 90% of cells in the INL and ONL of UPEN‐RB‐201 (*MYCN*‐amplified but *RB1* ^−/−^) were strongly positive for pRb; however, only about 70% of cells in the INL (Fig. 4M), and less than 5% in the ONL were ppRb immunoreactive (Fig. 4P). The INL of UPEN‐RB‐112 (*MYCN*‐low and *RB1* ^−/−^) had about 25% pRb‐positive cells, but only very few ppRb‐positive cells (Fig. 4S), and the ONL was essentially negative for both proteins (Fig. 4U) (Table 3).

Tumor sections of UPEN‐RB‐175 (*MYCN*‐amplified, *RB1* ^+/+^) stained strongly for pRb and ppRb (Fig. 4B,D), while the two *MYCN*‐amplified *RB1* ^+/−^ stained strongly with pRb antibody (Fig. 4F,J), but showed weaker staining of fewer cells with ppRb antibody (Fig. 4H,L). Interestingly, the well‐differentiated region of UPEN‐RB‐201 with many Flexner–Wintersteiner (FW) rosettes was mostly negative for both pRb and ppRb (Fig. 4O,R); however, the poorly differentiated section had scattered positive cells (Fig. 4N,Q). UPEN‐RB‐112 tumor was well differentiated with many FW rosettes and essentially negative for pRb and ppRb (Table S3 and Fig. 4). In all tumors, the immunoreactive pRb and ppRb proteins were primarily concentrated in the nucleus with very little staining of the cytoplasm.

**Figure 5:**
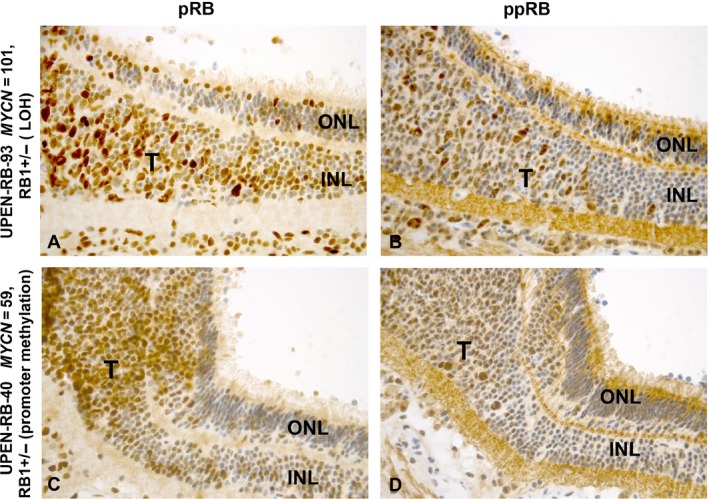
Transitional zone of the retina in two *MYCN*‐amplified, *RB1* ^+/−^ retinoblastomas, UPEN‐RB‐93 and UPEN‐RB‐40, immumostained with antibodies against pRb (A, C) and ppRb (B, D). The images show tumor cells migrating from the INL of the retina. The majority of cells in the region of the expanding tumors show moderate‐to‐strong reactivity for both pRb and ppRb compared to the adjacent INL which appears to be seeding the tumor, but where fewer cells are pRb‐positive and are mostly negative for ppRb. The staining pattern in the ONL of both pRb and ppRb appears comparable in this transition zone. Abbreviations: tumor (T), inner (INL) and outer (ONL) nuclear layer of the retina. (Original magnification ×40).

Figure 5 shows staining of the retinal section that seeds the retinoblastoma in two tumors with *MYCN* amplification, UPEN‐RB‐93 and UPEN‐RB‐40. With respect to pRb expression, there are relatively few scattered positive cells in both the ONL and INL; however, numerous darkly stained tumor cells can be seen originating from the adjacent weakly stained INL. Both tumors carry only one *RB1* mutation; so, some expression of pRb is to be expected. In contrast, there is very little expression of ppRb in the INL of either tumor but significant expression is seen in the adjacent tumor section for both tumors. These results appear to show that tumor cells expressing both pRb and ppRb proteins may originate in the retinal INL, and not from the ONL, However, it is also possible that these cells may originate elsewhere and migrate into the INL, or preferentially invade the INL at the edges of a large tumor.

## Discussion

Rushlow et al. reported a class of novel *RB1* ^+/+^ retinoblastoma with no genetic mutations in *RB1*, but having a high level of *MYCN* amplification (>29 copies) 2. This led to the hypothesis that high level *MYCN* amplification is sufficient for initiation of tumorigenesis of *RB1* ^+/+^ retinoblastoma. In this manuscript, we investigated the characteristics of *MYCN*‐amplified sporadic unilateral retinoblastoma and the mechanisms of inactivation of the *RB1* gene in these tumors.

Newly diagnosed, sporadic unilateral retinoblastoma is caused by somatic mutations in over 80% of cases, and by low penetrance germline mutations in the *RB1* gene in 15% of cases 4. The sensitivity of mutation detection in coding sequences of *RB1* is high, around 94% but not 100% 4. The current methods of mutation screening of *RB1* are limited to the promoter region, coding exons, and immediate flanking intronic regions, but not the entire introns which are known to harbor mutations 20, 21 and/or rearrangements including chromothripsis 7 that can also inactivate the *RB1* locus. Thus, based on current sensitivity of DNA‐based genetic testing, the presence of a subset of retinoblastoma without a detectable mutation in the *RB1* gene is not unexpected. Assuming a sensitivity for finding a coding sequence *RB1* mutation to be 94% 4, one would expect to observe only one wild‐type *RB1* ^+/+^ retinoblastoma among the 245 tested. However, we identified 13 (5.3%, *P* < 0.001) tumors without any *RB1* mutation.

We identified 18 of 245 retinoblastomas (7%) with a high *MYCN* copy number between 29 and 110 copies (Fig. 1). Of these, eight tumors carried two mutations in *RB1*, four were *RB1* ^+/−^, and six were *RB1* ^+/+^. This finding that 44% of *MYCN*‐amplified tumors carried two *RB1* mutations directly contrasts the observation by Rushlow et al. 2 who found no retinoblastomas with *MYCN* amplification among 93 tumors with two mutations in *RB1* (*RB1* ^−/−^). Further investigations into alternate mechanisms of inactivation of the *RB1* gene product demonstrated phosphorylation of pRb at S608 in tumors without coding sequence mutations in one or both alleles of *RB1* which is consistent with the observation that phosphorylation is the most common mechanism of pRb inactivation in most known cancers 22, 23.

*MYCN* amplification in retinoblastoma was first described by Lee et al. 24 showing that the *MYCN* gene was amplified 10–200‐fold in two primary retinoblastomas and in the Y79 cell line, but not in fibroblasts. Subsequently, Mairal et al. 14 assessed *MYCN* amplification karyotypically as double‐minute chromosomes and by PCR in four of 20 primary retinoblastomas. Further studies in a genetically engineered mouse model of human retinoblastoma demonstrated a 6–400‐fold amplification of *MYCN* in three of 16 aggressive retinoblastomas 25.

Amplification of *MYCN* has been described in about 20% of neuroblastoma where it identifies a highly aggressive subtype of the tumor 26, 27. In contrast, Lillington 28 found in a study of 25 primary retinoblastomas, that three tumors with *MYCN* amplification (>30 fold) were not associated with high‐risk histological features. In our series, retinoblastoma cases with high *MYCN* copy number were associated with an earlier age of onset that was significantly different from that of retinoblastoma without *MYCN* amplification (Fig. 2). However, there was no difference in the presence of high‐risk features between the two groups of tumors with and without the amplification.

In many cancers, the regulation of pRb becomes unbalanced, and elevated cyclin‐dependent kinase (CDK) activity prevents pRb from being an effective brake on cell cycle control. One of the potent CDK inhibitors is p27, a protein which is degraded by the protein, SKP2. In addition, SKP2 is a known target of *MYCN* amplification in neuroblastoma 29. The IHC staining results in our tumors indicate the absence of SKP2 protein in any significant amount and could signal high expression of p27 protein. However, the weak p27 immunoreactivity suggests that it is expressed in low levels in all *MYCN*‐amplified tumors, except the *RB1* ^−/−^ tumor, UPEN‐RB‐201, where it is highly expressed in both well and poorly differentiated areas of the tumor (Fig. S1, I, and J). Further comparison of p27 expression in UPEN‐RB‐201 and UPEN‐RB‐112 which is also *RB1* ^−/−^, but *MYCN*‐low suggests that p27 is up‐regulated in the presence of *MYCN* amplification, but not in *MYCN*‐low tumors in the absence of pRb. In essence, these observations rule out the activation of the MYCN‐SKP2‐p27 axis in three out of four *MYCN*‐amplified retinoblastomas. Since it is known that p27 is degraded upon progression into S‐phase when both pRb and p27 are phosphorylated 29, we investigated the phosphorylation status of pRb.

Alterations in pRb phosphorylation during late G1 phase allow the activation of E2F‐dependent transcription that drives the cell cycle and is needed for DNA replication. It has been shown that the alteration in the structure of pRb by phosphorylation at multiple residues, including S608/S612 30, 31 causes specific conformational changes and are sufficient for inhibition of interaction with the transactivating domain of E2F, while phosphorylation at S795 inhibits binding to the marked box (MB) domain of E2F1 29.

The observation that pRb is phosphorylated at S608 and/or S795 residue in the *MYCN*‐amplified retinoblastoma provides an important insight into how retinoblastoma develops without mutational knock‐out of the *RB1* gene which has conventionally been known as the rate‐limiting step in this process 1. In a recent paper, Liu et al. 32 have shown that following pRb loss, there is evidence for Myc‐dependent E2f3 accumulation and “rampant cell proliferation”. In commenting on these findings, Osorio 33 suggests that when functional pRb is not present in the cell, factors such as MYCN and E2Fs are “repurposed” resulting in unregulated cell proliferation.

Thus, in conclusion, in our series of 18 *MYCN*‐amplified retinoblastomas, 12 carry inactivating coding sequence mutations in the *RB1* gene and six do not. While inactivation of *RB1* by mutations has been classically associated with retinoblastoma, deregulation of the pRb pathway is very common in most types of human cancer 23, 24. Very few of these cancers carry mutations in the *RB1* gene, and pRb, is inactivated by phosphorylation in these nonretinoblastoma cancers. Similarly, in the retinoblastoma without coding sequence mutations, pRb, is inactivated by phosphorylation at various sites, including S608 and S795, which are two of the key residues that determine the interaction with E2F family of transcription factors 34. Hence, pRb inactivation, through mutation, phosphorylation or some other as yet unknown mechanism, is likely to be the initiating event in retinoblastoma tumorigenesis with or without *MYCN* amplification.

## Conflict of Interest

None declared.

## Supporting information


**Figure S1.** Expression of SKP2 and p27 in four *MYCN*‐amplified and one *MYCN*‐low retinoblastomas detected by immunohistological staining with anti‐SKP2 and anti‐p27^Kip1^ antibodies.

**Table S1.** *MYCN* copy number and *RB1* mutations found in 245 retinoblastomas.
**Table S2.** Histopathological features of 111 retinoblastomas.
**Table S3.** *RB1* gene mutations and high‐risk histological features in 18 tumors with *MYCN* amplification.
**Table S4.** IHC staining of five retinoblastoma tumors with antibodies specific for SKP2 and p27.

## References

[1] Knudson, A. G. 1971 Mutation and cancer: statistical study of retinoblastoma. Proc. Natl Acad. Sci. USA 68:820–823. 5279523 10.1073/pnas.68.4.820 PMC389051

[2] Rushlow, D. E. , B. M. Mol , J. Y. Kennett , S. Yee , S. Pajovic , B. L. Theriault , et al. 2013 Characterisation of retinoblastomas without RB1 mutations: genomic, gene expression, and clinical studies. Lancet Oncol. 14:327–334. 23498719 10.1016/S1470-2045(13)70045-7

[3] Chau, B. N. , and J. Y. J. Wang . 2003 Coordinated regulation of life and death by RB. Nat. Rev. Cancer 3:130–138. 12563312 10.1038/nrc993

[4] Chen, Z. , K. Moran , J. Richards‐Yutz , E. Toorens , D. Gerhart , T. Ganguly , et al. 2014 Enhanced Sensitivity for Detection of Low‐Level Germline Mosaic RB1 Mutations in Sporadic Retinoblastoma Cases Using Deep Semiconductor Sequencing. Hum. Mutat. 35:384–391. 24282159 10.1002/humu.22488 PMC4112364

[5] Ganguly, A. , K. E. Nichols , G. Grant , E. Rappaport , and C. Shields . 2009 Molecular karyotype of sporadic unilateral retinoblastoma tumors. Retina. 29:1002–1012. 19491728 10.1097/IAE.0b013e3181a0be05 PMC2722034

[6] Ganguly, A. , and C. L. Shields . 2010 Differential gene expression profile of retinoblastoma compared to normal retina. Mol. Vis. 16:1292–1303. 20664703 PMC2904042

[7] McEvoy, J. , P. Nagahawatte , D. Finkelstein , J. Richards‐Yutz , M. Valentine , J. Ma , et al. 2014 RB1 gene inactivation by chromothripsis in human retinoblastoma. Oncotarget. 5:438–450. 24509483 10.18632/oncotarget.1686 PMC3964219

[8] Nichols, K. E. , M. D. Houseknecht , L. Godmilow , G. Bunin , C. Shields , A. Meadows , et al. 2005 Sensitive multistep clinical molecular screening of 180 unrelated individuals with retinoblastoma detects 36 novel mutations in the RB1 gene. Hum. Mutat. 25:566–574. 15884040 10.1002/humu.20184

[9] Fokkema, I. F. A. C. , P. E. M. Taschner , G. C. P. Schaafsma , J. Celli , J. F. J. Laros , and J. T. den Dunnen . 2011 LOVD v.2.0: the next generation in gene variant databases. Hum. Mutat.. 32:557–563. Available from http://rb1-lovd.d-lohmann.de/home.php?select_db=RB1 21520333 10.1002/humu.21438

[10] Venneti, S. , P. Le , D. Martinez , K. W. Eaton , N. Shyam , K. L. Jordan‐Sciutto , et al. 2011 p16INK4A and p14ARF Tumor Suppressor Pathways Are Deregulated in Malignant Rhabdoid Tumors. J. Neuropathol. Exp. Neurol. 70:596–609. 21666498 10.1097/NEN.0b013e31822146ca PMC3145456

[11] Chen, D. , B. L. Gallie , and J. A. Squire . 2001 Minimal regions of chromosomal imbalance in retinoblastoma detected by comparative genomic hybridization. Cancer Genet. Cytogenet. 129:57–63. 11520568 10.1016/s0165-4608(01)00427-7

[12] Corson, T. W. , and B. L. Gallie . 2007 One hit, two hits, three hits, more? Genomic changes in the development of retinoblastoma. Genes Chromosom. Cancer 46:617–634. 17437278 10.1002/gcc.20457

[13] Herzog, S. , D. R. Lohmann , K. Buiting , A. Schuler , B. Horsthemke , H. Rehder , et al. 2001 Marked differences in unilateral isolated retinoblastomas from young and older children studied by comparative genomic hybridization. Hum. Genet. 108:98–104. 11281459 10.1007/s004390000450

[14] Mairal, A. , E. Pinglier , E. Gilbert , M. Peter , P. Validire , L. Desjardins , et al. 2000 Detection of chromosome imbalances in retinoblastoma by parallel karyotype and CGH analyses. Genes Chromosom. Cancer 28:370–379. 10862045

[15] van der Wal, J. E. , M. A. Hermsen , H. J. Gille , N. Y. Schouten‐Van Meeteren , A. C. Moll , S. M. Imhof , et al. 2003 Comparative genomic hybridisation divides retinoblastomas into a high and a low level chromosomal instability group. J. Clin. Pathol. 56:26–30. 12499428 10.1136/jcp.56.1.26 PMC1769844

[16] Zielinski, B. , S. Gratias , G. Toedt , F. Mendrzyk , D. E. Stange , B. Radlwimmer , et al. 2005 Detection of chromosomal imbalances in retinoblastoma by matrix‐based comparative genomic hybridization. Genes Chromosom. Cancer 43:294–301. 15834944 10.1002/gcc.20186

[17] Thériault, B. L. , H. Dimaras , B. L. Gallie , and T. W. Corson . 2014 The genomic landscape of retinoblastoma: a review. Clin. Exp. Ophthalmol. 42:33–52. 24433356 10.1111/ceo.12132 PMC3896868

[18] Retinoblastoma . 2010 Retinoblastoma. pp. 562–563 *in* Edge S. B., Byrd D. R., Compton C. C., Fritz A. G., Greene F. L., eds. AJCC Cancer Staging Manual, 7th ed. Springer, New York, NY.

[19] Kaliki, S. , C. L. Shields , D. Rojanaporn , S. Al‐Dahmash , J. P. McLaughlin , J. A. Shields , et al. 2013 High‐risk retinoblastoma based on International Classification of Retinoblastoma: analysis of 519 enucleated eyes. Ophthalmology 120:997–1003. 23399379 10.1016/j.ophtha.2012.10.044

[20] Dehainault, C. , D. Michaux , S. Pages‐Berhouet , V. Caux‐Moncoutier , F. Doz , L. Desjardins , et al. 2007 A deep intronic mutation in the RB1 gene leads to intronic sequence exonisation. Eur. J. Hum. Genet. 15:473–477. 17299438 10.1038/sj.ejhg.5201787

[21] Zhang, K. , I. Nowak , D. Rushlow , B. L. Gallie , and D. R. Lohmann . 2008 Patterns of missplicing caused by RB1 gene mutations in patients with retinoblastoma and association with phenotypic expression. Hum. Mutat. 29:475–484. 18181215 10.1002/humu.20664

[22] Giacinti, C. , and A. Giordano . 2006 RB and cell cycle progression. Oncogene 25:5220–5227. 16936740 10.1038/sj.onc.1209615

[23] Harbour, J. W. , and D. C. Dean . 2000 The Rb/E2F pathway: expanding roles and emerging paradigms. Genes Dev. 14:2393–2409. 11018009 10.1101/gad.813200

[24] Lee, W. H. , A. L. Murphree , and W. F. Benedict . 1984 Expression and amplification of the N‐myc gene in primary retinoblastoma. Nature 309:458–460. 6728001 10.1038/309458a0

[25] MacPherson, D. , K. Conkrite , M. Tam , S. Mukai , D. Mu , and T. Jacks . 2007 Murine bilateral retinoblastoma exhibiting rapid‐onset, metastatic progression and N‐myc gene amplification. The EMBO Journal. 26:784–794. 17235288 10.1038/sj.emboj.7601515 PMC1794380

[26] Muth, D. , S. Ghazaryan , I. Eckerle , E. Beckett , C. Pöhler , J. Batzler , et al. 2010 Transcriptional repression of SKP2 is impaired in MYCN‐amplified neuroblastoma. Cancer Res. 70:3791–3802. 20424123 10.1158/0008-5472.CAN-09-1245

[27] Westermann, F. , K.‐O. Henrich , J. S. Wei , W. Lutz , M. Fischer , R. König , et al. 2007 High Skp2 expression characterizes high‐risk neuroblastomas independent of MYCN status. Clin. Cancer Res. 13:4695–4703. 17652624 10.1158/1078-0432.CCR-06-2818

[28] Lillington, D. M. , L. K. Goff , J. E. Kingston , Z. Onadim , E. Price , P. Domizio , et al. 2002 High level amplification of N‐MYC is not associated with adverse histology or outcome in primary retinoblastoma tumours. Br. J. Cancer 87:779–782. 12232763 10.1038/sj.bjc.6600532 PMC2364265

[29] Rubin, S. M. 2013 Deciphering the retinoblastoma protein phosphorylation code. Trends Biochem. Sci. 38:12–19. 23218751 10.1016/j.tibs.2012.10.007 PMC3529988

[30] Burke, J. R. , G. L. Hura , and S. M. Rubin . 2012 Structures of inactive retinoblastoma protein reveal multiple mechanisms for cell cycle control. Genes Dev. 26:1156–1166. 22569856 10.1101/gad.189837.112 PMC3371405

[31] Takaki, T. , K. Fukasawa , I. Suzuki‐Takahashi , K. Semba , M. Kitagawa , Y. Taya , et al. 2005 Preferences for phosphorylation sites in the retinoblastoma protein of D‐type cyclin–dependent kinases, Cdk4 and Cdk 6, in Vitro. J. Biochem. 137:381–386. 15809340 10.1093/jb/mvi050

[32] Liu, H. , X. Tang , A. Srivastava , T. Pecot , P. Daniel , B. Hemmelgarn , et al. 2015 Redeployment of Myc and E2f1‐3 drives Rb‐deficient cell cycles. Nat. Cell Biol. 17:1036–1048. 26192440 10.1038/ncb3210 PMC4526313

[33] Osorio, J. 2015 Cell cycle: repurposing MYC and E2F in the absence of RB. Nat. Rev. Mol. Cell Biol. 16:516–517. 10.1038/nrm4044 26265406

[34] Heilmann, A. M. F. , and N. J. Dyson . 2012 Phosphorylation puts the pRb tumor suppressor into shape. Genes Dev. 26:1128–1130. 22661226 10.1101/gad.195552.112 PMC3371403

