# Supplementary figures and images for: Phosphorylation of pRb: mechanism for RB pathway inactivation in MYCN‐amplified retinoblastoma
Source: Cancer Med. 2017 Feb 17;6(3):619–30. doi: 10.1002/cam4.1010 (PMC5345671; doi:10.1002/cam4.1010)

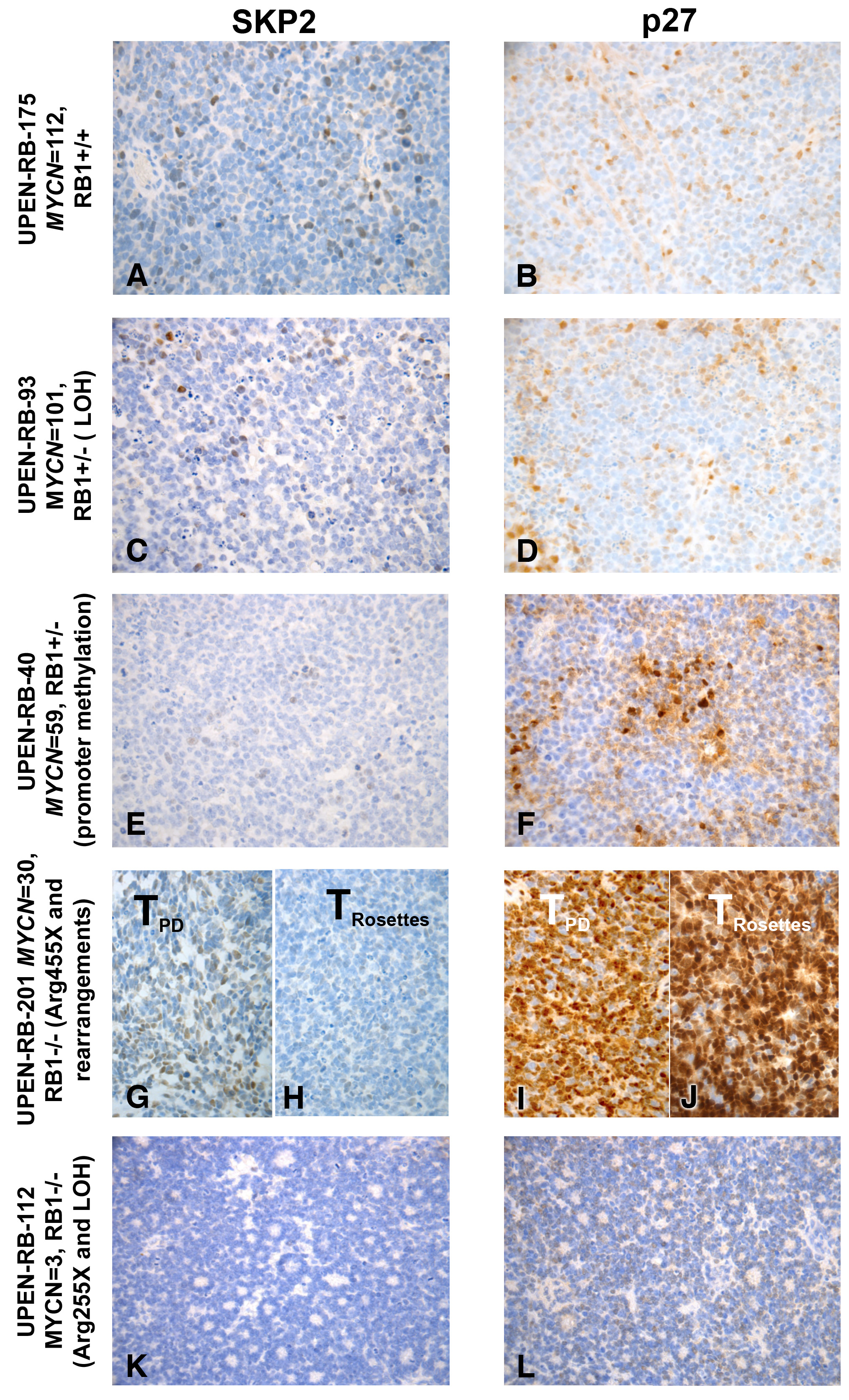

Supplement: Supplementary file 1 — Figure S1. Expression of SKP2 and p27 in four MYCN‐amplified and one MYCN‐low retinoblastomas detected by immunohistological staining with anti‐SKP2 and anti‐p27Kip1 antibodies. [file CAM4-6-619-s001.jpg]
