# Supplementary material for: Phosphorylation of pRb: mechanism for RB pathway inactivation in MYCN‐amplified retinoblastoma
Source: Cancer Med. 2017 Feb 17;6(3):619–30. doi: 10.1002/cam4.1010 (PMC5345671; doi:10.1002/cam4.1010)
Supplement: Supplementary file 2 — Table S1. MYCN copy number and RB1 mutations found in 245 retinoblastomas. Table S2. Histopathological features of 111 retinoblastomas. Table S3. RB1 gene mutations and high‐risk histological features in 18 tumors with MYCN amplification. Table S4. IHC staining of five retinoblastoma tumors with antibodies specific for SKP2 and p27. [file CAM4-6-619-s002.docx]

Supporting Table 1. *MYCN* copy number and *RB1* mutations found in 245 retinoblastomas.

| **UPEN-RB_ID** | **Age at diagnosis** | **Gender** | ***MYCN* Copy Number** | ***RB1* mutation status** | ***RB1* mutation #1** | ***RB1* mutation #2** | **Figure 1 notation** |  |
| --- | --- | --- | --- | --- | --- | --- | --- | --- |
| **UPEN-RB-125** | 1 | female | **128** | RB1^+/+^ | none identified | none identified | 245 |  |
| **UPEN-RB-175** | 10 | female | **112** | RB1^+/+^ | none identified | none identified | 244 |  |
| **UPEN-RB-127** | 13 | male | **108** | RB1^+/+^ | none identified | none identified | 243 |  |
| **UPEN-RB-93** | 5 | male | **101** | RB1^+/-^ | LOH | none identified | 242 |  |
| **^1^UPEN-RB-07** | 6 | female | **84** | RB1^+/+^ | none identified | none identified | 241 |  |
| **UPEN-RB-200** | 33 | male | **74** | RB1^-/-^ | ex_1 g.2101 C>T; c.42C>T (p.Ala14Ala) | promoter methylation | 240 |  |
| **UPEN-RB-198** | 4 | female | **69** | RB1^+/-^ | complex rearrangement | none identified | 239 |  |
| **UPEN-RB-176** | 7 | male | **65** | RB1^+/+^ | none identified | none identified | 238 |  |
| **UPEN-RB-114** | 5 | female | **64** | RB1^+/+^ | none identified | none identified | 237 |  |
| **^1^UPEN-RB-40** | 9 | male | **59** | RB1^+/-^ | promoter methylation | none identified | 236 |  |
| **UPEN-RB-147** | 28 | male | **51** | RB1^-/-^ | ex_3 g.39485_39486delTG; c.304-305del (p.Cys102Tyrfs*7) | in_13 g.73870 G>T c.1332 + 1G>T | 235 |  |
| **UPEN-RB-199** | 39 | female | **44** | RB1^-/-^ | ex_17 g.78117delT; c.1533delT (p.Asp511Aspfs*7) | dup exon 1 | 234 |  |
| **^1^UPEN-RB-05** | 25 | female | **42** | RB1^-/-^ | ex_15 g.76898C>T; c.1399C>T (p.Arg467*) | LOH | 233 |  |
| **UPEN-RB-135** | 26 | male | **39** | RB1^-/-^ | ex_4 g.42008dupT; c.463dupT (p.Tyr155Leufs*2) | ex_17 g.78185dupA c.1601dupA (p.Ser534SLysfs*21) | 232 |  |
| **^1^UPEN-RB-45** | 6 | male | **38** | RB1^-/-^ | LOH | LOH | 231 |  |
| **UPEN-RB-153** | 40 | male | **35** | RB1^-/-^ | ex_8 g.59695 C>T; c.763C>T (p.Arg255*) | LOH | 230 |  |
| **UPEN-RB-201** | 9 | male | **30** | RB1^-/-^ | ex_14 g.76460C>T; c.1363C>T (p.Arg455*) | complex rearrangement | 229 |  |
| **UPEN-RB-115** | 24 | female | **30** | RB1^+/-^ | ex_8 g.59695C>T; c.763C>T (p.Arg255*) | none identified | 228 |  |
| **UPEN-RB-150** | 32 | male | 19 | RB1^-/-^ | ex_17 g.78158C>G; c.1574C>G (p.Ala525Gly) | ex_20 g.156705C>T c.1973C>T (p.Ala658Val) | 227 |  |
| **UPEN-RB-192** | 22 | female | 18 | RB1^-/-^ | ex_17 g.78238C>T; c.1654C>T (p.Arg552*) | ex_19 g.153352dupA c.1959dupA (p.Val654Serfs*14) | 226 |  |
| **^1^UPEN-RB-47** | 48 | male | 17 | RB1^-/-^ | ex_2 g.5503delA; c.217delA (p.Arg73Glufs*3) | ex_9 g.61752insA c.844insA (p.Asn295Lysfs*14) | 225 |  |
| **^1^UPEN-RB-31** | 18 | female | 17 | RB1^+/-^ | del exons 3-20 | none identified | 224 |  |
| **UPEN-RB-215** | 24 | male | 16 | RB1^-/-^ | ex_3 g.39551_39552delAT; c.371-372del (p.Ile124Argfs*6) | LOH | 223 |  |
| **UPEN-RB-236** | 29 | male | 16 | RB1^-/-^ | del exons 1-27 | promoter methylation | 222 |  |
| **UPEN-RB-159** | 28 | female | 15 | RB1^-/-^ | ex_11 g.65373insA; c.1059insA (p.Gln354Thrfs*7) | LOH | 221 |  |
| **UPEN-RB-163** | 20 | male | 15 | RB1^-/-^ | ex_8 g.59727delA; c.795delA (p.Lys265Asnfs*2) | ex_14 g.76430C>T c.1333C>T (p.Arg445*) | 220 |  |
| **UPEN-RB-241** | 47 | female | 14 | RB1^-/-^ | promoter methylation | LOH | 219 |  |
| **UPEN-RB-02** | 31 | male | 13 | RB1^-/-^ | ex_8 g.59695C>T; c.763C>T (p.Arg255*) | LOH | 218 |  |
| **UPEN-RB-212** | 36 | female | 11 | RB1^-/-^ | ex_15 g.76898C>T; c.1399C>T (p.Arg467*) | LOH | 217 |  |
| **UPEN-RB-142** | 19 | female | 10 | RB1^-/-^ | ex_10 g.64348C>T; c.958C>T (p.Arg320*) | LOH | 216 |  |
| **UPEN-RB-193** | NA | female | 10 | RB1^-/-^ | ex_7 g.56904_56905delTA; c.659_660delTA (p.Val222Profs*1) | LOH | 215 |  |
| **^1^UPEN-RB-41** | 19 | female | 9 | RB1^-/-^ | del exons 1-27 | del exons 1-27 | 214 |  |
| **^1^UPEN-RB-18** | 13 | female | 8 | RB1^-/-^ | promoter methylation | LOH | 213 |  |
| **UPEN-RB-95** | NA | female | 8 | RB1^-/-^ | promoter methylation | LOH | 212 |  |
| **UPEN-RB-132** | 38 | male | 8 | RB1^-/-^ | ex_16 g.77051T>C; c.1472T>C (p.Leu491Pro) | LOH | 211 |  |
| **UPEN-RB-126** | 18 | male | 8 | RB1^-/-^ | promoter methylation | LOH | 210 |  |
| **UPEN-RB-81** | 33 | female | 8 | RB1^-/-^ | ex_9 g.61744_161757del14; c.876_889del14 (p.Phe293Thrfs*11) | LOH | 209 |  |
| **UPEN-RB-84** | 37 | male | 7 | RB1^-/-^ | promoter methylation | LOH | 208 |  |
| **UPEN-RB-118** | 25 | male | 7 | RB1^-/-^ | ex_17 g.78238C>T; c.1654C>T (p.Arg552*) | ex_18 g.150025C>T c.1723C>T (p.Gln575*) | 207 |  |
| **UPEN-RB-104** | 60 | female | 7 | RB1^-/-^ | dup exons 1-27 | promoter methylation | 206 |  |
| **UPEN-RB-80** | 30 | female | 7 | RB1^+/-^ | LOH | none identified | 205 |  |
| **UPEN-RB-244** | 38 | female | 6 | RB1^-/-^ | ex_15 g.76898C>T; c.1399C>T (p.Arg467*) | LOH | 204 |  |
| **UPEN-RB-206** | 38 | female | 6 | RB1^-/-^ | del exons 1-27 | del exons 1-27 | 203 |  |
| **UPEN-RB-111** | 32 | male | 6 | RB1^-/-^ | del exons 15,16 | promoter methylation | 202 |  |
| **UPEN-RB-157** | 18 | female | 6 | RB1^-/-^ | in_12 g.70330G>A; c.1215 + 1G>A | ex_20 g.156705C>A c.1973C>A (p.Ala658Asp) | 201 |  |
| **^1^UPEN-RB-49** | 60 | male | 6 | RB1^-/-^ | ex_8 g.59695C>T; c.763C>T (p.Arg255*) | ex_19 g.153332_153333delCT c.1939-1940delCT (p.Leu647Phefs*5) | 200 |  |
| **UPEN-RB-133** | 24 | male | 6 | RB1^-/-^ | in_10 g.64440G>A; c.1049 + 1G>A | LOH | 199 |  |
| **UPEN-RB-148** | 42 | female | 6 | RB1^-/-^ | complex rearrangement | promoter methylation | 198 |  |
| **UPEN-RB-89** | 60 | female | 6 | RB1^-/-^ | ex_17 g.78250C>T; c.1666C>T (p.Arg556*) | LOH | 197 |  |
| **UPEN-RB-253** | 35 | female | 5 | RB1^-/-^ | ex_12 g.70314_70318dupGATTT; c.1200-1204dupGATTT (p.Ille401Ilefs*2) | LOH | 196 |  |
| **UPEN-RB-138** | 56 | female | 5 | RB1^-/-^ | del exons 7 and 21 | del exons 7 and 22 | 195 |  |
| **UPEN-RB-254** | 45 | female | 5 | RB1^+/-^ | ex_9 g.61788C>T; c.920C>T (p.Thr307Ile) | none identified | 194 |  |
| **UPEN-RB-172** | 24 | male | 5 | RB1^-/-^ | ex_21 g.160832G>T; c.2209G>T (p.Glu737*) | LOH | 193 |  |
| **UPEN-RB-75** | 27 | female | 5 | RB1^-/-^ | ex_10 g.64348C>T; c.958C>T (p.Arg320*) | promoter methylation | 192 |  |
| **UPEN-RB-249** | 24 | female | 5 | RB1^-/-^ | in_13 g.76429G>A; c.1333 - 1G>A | ex_15 g.76898C>T c.1399C>T (p.Arg467*) | 191 |  |
| **UPEN-RB-229** | 18 | female | 5 | RB1^-/-^ | ex_2 g.5505_5506dupAG; c.219-220dupAG (p.Ala74Glufs*4) | in_10 g.64440G>T c.1049 + 1G>T | 190 |  |
| **UPEN-RB-179** | 35 | female | 5 | RB1^-/-^ | ex_14 g.76460C>T; c.1363C>T (p.Arg455*) | LOH | 189 |  |
| **UPEN-RB-92** | 60 | male | 5 | RB1^+/-^ | in_13 g.73870G>A; c.1332 + 1G>T | none identified | 188 |  |
| **UPEN-RB-62** | 39 | female | 5 | RB1^-/-^ | in_21 g.161984T>A; c.2212 - 13T>A | LOH | 187 |  |
| **^1^UPEN-RB-50** | 24 | female | 5 | RB1^-/-^ | ex_10 g.64348C>T; c.958C>T (p.Arg320*) | LOH | 186 |  |
| **UPEN-RB-154** | 3 | male | 5 | RB1^-/-^ | ex_8 g.59695C>T; c.763C>T (p.Arg255*) | LOH | 185 |  |
| **UPEN-RB-169** | 76 | female | 5 | RB1^-/-^ | ex_1 g.2137_2139delGCC c.78_80del (p.Pro29del) | ex_23 g.162331delG; c.2453delG (p.Gly818Valfs*7) | 184 |  |
| **UPEN-RB-165** | 36 | male | 5 | RB1^-/-^ | deletion/rearragement | LOH | 183 |  |
| **UPEN-RB-96** | 23 | male | 5 | RB1^-/-^ | ex_17 g.78271delT; c.1687delT (p.Trp563Glyfs*47) | promoter methylation | 182 |  |
| **UPEN-RB-140** | 38 | female | 5 | RB1^-/-^ | ex_11 g.65374_65375delCA; c.1060-1061delCA (p.Gln354Glufs*7) | ex_20 g.156705C>A c.1973C>A (p.Ala658Asp) | 181 |  |
| **^1^UPEN-RB-33** | 73 | female | 5 | RB1^-/-^ | in_13 g.76426G>A; c.1333 - 4G>A | LOH | 180 |  |
| **UPEN-RB-83** | 11 | male | 5 | RB1^-/-^ | ex_15 g.76898C>T; c.1399C>T (p.Arg467*) | LOH | 179 |  |
| **UPEN-RB-100** | 23 | female | 5 | RB1^+/-^ | LOH | none identified | 178 |  |
| **UPEN-RB-186** | 26 | male | 5 | RB1^-/-^ | ex_8 g.59695C>T; c.763C>T (p.Arg255*) | LOH | 177 |  |
| **^1^UPEN-RB-04** | 17 | male | 5 | RB1^-/-^ | promoter methylation | LOH | 176 |  |
| **UPEN-RB-247** | 6 | female | 4 | RB1^-/-^ | ex_17 g.78238C>T; c.1654C>T (p.Arg552*) | LOH | 175 |  |
| **UPEN-RB-205** | 27 | male | 4 | RB1^-/-^ | in_20 g.160729G>C; c.2107 - 1G>C | LOH | 174 |  |
| **UPEN-RB-164** | 24 | female | 4 | RB1^-/-^ | ex_14 g.76460C>T; c.1363C>T (p.Arg455*) | LOH | 173 |  |
| **^1^UPEN-RB-03** | 4 | male | 4 | RB1^-/-^ | complex rearrangement | LOH | 172 |  |
| **UPEN-RB-73** | 25 | male | 4 | RB1^-/-^ | ex_18 g.150038_150047del10; c.1736_1745del10 (p.Arg579Glnfs*29) | promoter methylation | 171 |  |
| **UPEN-RB-70** | 28 | male | 4 | RB1^-/-^ | ex_20 g.156731_156745 del15; c.1999_2013del15 (p.Glu667_Ser671del) | LOH | 170 |  |
| **UPEN-RB-173** | 15 | female | 4 | RB1^+/-^ | ex_2 g.5505_5506delAG; c.219-220delAG (p.Arg73Serfs*36) | none identified | 169 |  |
| **UPEN-RB-234** | 125 | male | 4 | RB1^-/-^ | ex_6 g.45834_45835delAA; c576-577 (p.Lys192Serfs*9) | LOH | 168 |  |
| **UPEN-RB-166** | 32 | male | 4 | RB1^-/-^ | ex_14 g.76443G>T; c.1346G>T (p.Gly449Val) | in_25 g.173851T>C c.2663 + 2T>C | 167 |  |
| **^1^UPEN-RB-20** | 25 | male | 4 | RB1^-/-^ | ex_12 g.70262insC; c.1148insC (p.Gln383Profs*11) | in_12 g.70330G>A c.1216 + 1G>A | 166 |  |
| **UPEN-RB-105** | 48 | female | 4 | RB1^-/-^ | ex_11 g.65386C>T; c.1072C>T (p.Arg358*) | LOH | 165 |  |
| **UPEN-RB-71** | 30 | female | 4 | RB1^-/-^ | ex_8 g.59695 C>T; c.763C>T (p.Arg255*) | LOH | 164 |  |
| **UPEN-RB-230** | 24 | male | 4 | RB1^-/-^ | ex_25 g.173849G>T; c.2663G>A (p.Ser888Ile) | LOH | 163 |  |
| **UPEN-RB-78** | 26 | male | 4 | RB1^-/-^ | ex_14 g.76460C>T; c.1363C>T (p.Arg455*) | LOH | 162 |  |
| **UPEN-RB-82** | 21 | female | 4 | RB1^+/-^ | LOH | none identified | 161 |  |
| **UPEN-RB-61** | 19 | female | 4 | RB1^+/-^ | promoter methylation | none identified | 160 |  |
| **UPEN-RB-252** | 27 | male | 4 | RB1^-/-^ | ex_3 g.39486_39487delGT; c.305-306del (p.Cys102Tyrfs*7) | ex_18 g.150037C>T c.1735C>T (p.Arg579*) | 159 |  |
| **UPEN-RB-250** | 39 | male | 4 | RB1^-/-^ | ex_14 g.76430C>T; c.1333C>T (p.Arg445*) | LOH | 158 |  |
| **UPEN-RB-151** | 50 | male | 4 | RB1^-/-^ | ex_10 g.64348C>T; c.958C>T (p.Arg320*) | ex_10 g.64414delA c.1024delA (p.Thr342Leufs*7) | 157 |  |
| **UPEN-RB-72** | 24 | female | 4 | RB1^-/-^ | ex_10 g.64348 C>T; c.958C>T (p.Arg320*) | ex_23 g.162237 C>T c.2359C>T (p.Arg787*) | 156 |  |
| **UPEN-RB-117** | 40 | female | 4 | RB1^-/-^ | promoter methylation | LOH | 155 |  |
| **UPEN-RB-74** | 54 | female | 4 | RB1^-/-^ | ex_11 g.65386 C>T; c.1072C>T (p.Arg358*) | LOH | 154 |  |
| **UPEN-RB-251** | 27 | female | 4 | RB1^-/-^ | ex_11 g.65386C>T; c.1072C>T (p.Arg358*) | LOH | 153 |  |
| **UPEN-RB-245** | 14 | female | 4 | RB1^-/-^ | ex_23 g.162248C>A; c.2370C>A (p.Tyr790*) | promoter methylation | 152 |  |
| **UPEN-RB-239** | 36 | male | 4 | RB1^-/-^ | ex_1 g.2184_2191del8; c.125-132del8 (p.Leu42Argfs*3) | complex rearrangement | 151 |  |
| **UPEN-RB-235** | 24 | male | 4 | RB1^-/-^ | promoter methylation | LOH | 150 |  |
| **UPEN-RB-232** | 32 | female | 4 | RB1^-/-^ | del exons 1-27 | del exons 1-27 | 149 |  |
| **UPEN-RB-231** | 10 | male | 4 | RB1^-/-^ | del exon 1-27 | LOH | 148 |  |
| **UPEN-RB-228** | 11 | male | 4 | RB1^-/-^ | promoter methylation | LOH | 147 |  |
| **UPEN-RB-196** | 38 | female | 4 | RB1^-/-^ | ex_1 g.2195_2196AG>CT; c.136-136AG>CT (p.Arg46Leu) | ex_11 g.65423delC c.1109delC (p.Pro370Leufs*9) | 146 |  |
| **UPEN-RB-181** | 33 | male | 4 | RB1^-/-^ | ex_14 g.76430C>T; c.1333C>T (p.Arg445*) | ex_18 g.150040G>T c.1738G>T (p.Glu580*) | 145 |  |
| **UPEN-RB-155** | 1 | male | 4 | RB1^-/-^ | ex_17 g.78271T>C; c.1687T>C (p.Trp563Arg) | promoter methylation | 144 |  |
| **^1^UPEN-RB-44** | 50 | male | 4 | RB1^-/-^ | ex_14 g.76460C>T; c.1363C>T (p.Arg455*) | dup exon 1 | 143 |  |
| **^1^UPEN-RB-39** | 34 | male | 4 | RB1^-/-^ | ex_5 g.44682_44685delTATA; c.515-515delTATA (p.Ile172Ilefs*1) | LOH | 142 |  |
| **^1^UPEN-RB-37** | NA | ?? | 4 | RB1^-/-^ | in_12 g.70330G>A; c.1215 + 1G>A | LOH | 141 |  |
| **UPEN-RB-109** | 31 | male | 4 | RB1^-/-^ | ex_18 g.150037C>T; c.1735C>T (p.Arg579*) | LOH | 140 |  |
| **^1^UPEN-RB-15** | 22 | male | 4 | RB1^-/-^ | ex_22 g.162032T>A; c.2247T>A (p.Tyr749*) | LOH | 139 |  |
| **^1^UPEN-RB-01** | 18 | female | 4 | RB1^-/-^ | ex_15 g.76898C>T; c.1399C>T (p.Arg467*) | ex_20 g.156775G>A c.2043G>A (p.Trp681*) | 138 |  |
| **UPEN-RB-54** | 14 | male | 4 | RB1^-/-^ | promoter methylation | LOH | 137 |  |
| **UPEN-RB-88** | 7 | female | 4 | RB1^+/-^ | LOH | none identified | 136 |  |
| **UPEN-RB-216** | 7 | female | 4 | RB1^-/-^ | ex_24 g.170380dupT; c.2498dupT (p.Ser834Ilefs*4) | LOH | 135 |  |
| **UPEN-RB-177** | 24 | female | 4 | RB1^-/-^ | ex_16 g.77035_77036delTT; c.1456_1457delTT (p.Leu486Ilefs*6) | ex_21 g.160787A>T c.2164A>T (p.Lys722*) | 134 |  |
| **UPEN-RB-168** | 36 | male | 4 | RB1^-/-^ | ex_17 g.78238C>T; c.1654C>T (p.Arg552*) | in_23 g.170371G>A c.2490 - 1G>A | 133 |  |
| **^1^UPEN-RB-42** | 35 | male | 4 | RB1^-/-^ | in_12 g.70330G>A; c.1215 + 1G>A | ex_14 g.76460C>T c.1363C>T (p.Arg455*) | 132 |  |
| **^1^UPEN-RB-26** | 26 | female | 4 | RB1^-/-^ | ex_23 g.162237C>T; c.2359C>T (p.Arg787*) | promoter methylation | 131 |  |
| **UPEN-RB-77** | 10 | female | 4 | RB1^-/-^ | promoter methylation | LOH | 130 |  |
| **UPEN-RB-64** | 5 | female | 4 | RB1^-/-^ | ex_11 g.65386C>T; c.1072C>T (p.Arg358*) | LOH | 129 |  |
| **UPEN-RB-55** | 60 | male | 4 | RB1^-/-^ | ex_18 g.150037C>T; c.1735C>T (p.Arg579*) | LOH | 128 |  |
| **UPEN-RB-56** | 24 | female | 4 | RB1^+/+^ | none identified | none identified | 127 |  |
| **UPEN-RB-202** | 36 | female | 4 | RB1^-/-^ | del of exons 3-20 | del of exons 3-20 | 126 |  |
| **UPEN-RB-190** | 24 | female | 4 | RB1^-/-^ | promoter methylation | LOH | 125 |  |
| **UPEN-RB-152** | 20 | male | 4 | RB1^-/-^ | in_12 g.70330G>A; c.1215 + 1G>A | promoter methylation | 124 |  |
| **UPEN-RB-67** | 2 | female | 4 | RB1^-/-^ | promoter methylation | LOH | 123 |  |
| **UPEN-RB-243** | 20 | female | 4 | RB1^-/-^ | ex_7 g.56855G>T; c.610G>T (p.Glu204*) | ex_14 g.76460C>T c.1363C>T (p.Arg455*) | 122 |  |
| **UPEN-RB-238** | 35 | male | 4 | RB1^-/-^ | promoter methylation | LOH | 121 |  |
| **UPEN-RB-208** | 8 | male | 4 | RB1^-/-^ | promoter methylation | LOH | 120 |  |
| **UPEN-RB-189** | 24 | male | 4 | RB1^-/-^ | ex_23 g.162237C>T; c.2359C>T (p.Arg787*) | promoter methylation | 119 |  |
| **UPEN-RB-161** | 40 | male | 4 | RB1^-/-^ | del exons 1-27 | promoter methylation | 118 |  |
| **UPEN-RB-160** | 12 | female | 4 | RB1^-/-^ | ex_17 g.78131G>A; c.1547G>A (p.Trp516*) | ex_18 g.150037delC c.1735delC (p.Arg579Glufs*32) | 117 |  |
| **UPEN-RB-149** | 30 | female | 4 | RB1^-/-^ | ex_12 g.70278_20296del19; c.1164_1182del19 (p.Leu389Asnfs*5) | LOH | 116 |  |
| **UPEN-RB-69** | 9 | female | 4 | RB1^-/-^ | promoter methylation | LOH | 115 |  |
| **UPEN-RB-58** | 24 | female | 4 | RB1^+/-^ | LOH | none identified | 114 |  |
| **UPEN-RB-182** | 2 | female | 4 | RB1^-/-^ | ex_20 g.156727_156728delTT; c.1995_1996delTT (p.Cys666*) | LOH | 113 |  |
| **^1^UPEN-RB-48** | 22 | female | 4 | RB1^-/-^ | ex_10 g.64348C>T; c.958C>T (p.Arg320*) | ex_18 g.150022A>T c.1720A>T (p.Lys574*) | 112 |  |
| **UPEN-RB-145** | 23 | male | 4 | RB1^-/-^ | ex_11 g.65386C>T; c.1072C>T (p.Arg358*) | del exons 9-12 | 111 |  |
| **UPEN-RB-141** | 25 | female | 4 | RB1^-/-^ | in_12 g.73752G>A; c.1216 - 1G>A | ex_20 g.156713C>T c.1981C>T (p.Arg661Trp) | 110 |  |
| **UPEN-RB-131** | 6 | female | 4 | RB1^-/-^ | ex_14 g.76460C>T; c.1363C>T (p.Arg455*) | promoter methylation | 109 |  |
| **UPEN-RB-130** | 50 | male | 4 | RB1^-/-^ | in_12 g.70330G>A; c.1215 + 1G>A | promoter methylation | 108 |  |
| **^1^UPEN-RB-16** | 41 | male | 4 | RB1^-/-^ | ex_4 g.41954G>T; c.409G>T (p.Glu137*) | ex_17 g.78279_78282delAGTA c.1695_1698delAGTA (splice ) | 107 |  |
| **UPEN-RB-103** | 8 | male | 4 | RB1^-/-^ | promoter methylation | LOH | 106 |  |
| **UPEN-RB-87** | 30 | female | 4 | RB1^-/-^ | promoter methylation | LOH | 105 |  |
| **UPEN-RB-180** | 22 | female | 4 | RB1^+/-^ | LOH | none identified | 104 |  |
| **UPEN-RB-227** | 10 | male | 3 | RB1^-/-^ | del exons 1-27 | del exons 1-27 | 103 |  |
| **UPEN-RB-139** | 28 |  | 3 | RB1^-/-^ | del exon 1-27 | LOH | 102 |  |
| **^1^UPEN-RB-13** | 16 | female | 3 | RB1^-/-^ | in_14 g.76490A>G; c.1389 + 4A>G | LOH | 101 |  |
| **UPEN-RB-90** | 9 | male | 3 | RB1^-/-^ | ex_21 g.160740G>A; c.2117G>A (p.Cys706Tyr) | LOH | 100 |  |
| **UPEN-RB-53** | 6 | female | 3 | RB1^-/-^ | ex_14 g.76460C>T; c.1363C>T (p.Arg455*) | LOH | 99 |  |
| **UPEN-RB-51** | 10 | female | 3 | RB1^-/-^ | ex_2 g.5437G>T; c.151G>T (p.Glu51*) | LOH | 98 |  |
| **UPEN-RB-223** | 136 | female | 3 | RB1^-/-^ | ex_20 g.156824_156825delAG; c.3092_2093delAG (p.Arg698Alafs*2) | LOH | 97 |  |
| **UPEN-RB-162** | 34 | female | 3 | RB1^-/-^ | del of exons 1-27 | del of exons 1-27 | 96 |  |
| **^1^UPEN-RB-09** | 1 | female | 3 | RB1^-/-^ | promoter methylation | LOH | 95 |  |
| **UPEN-RB-60** | 3 | female | 3 | RB1^-/-^ | promoter methylation | LOH | 94 |  |
| **UPEN-RB-183** | 43 | male | 3 | RB1^+/-^ | del exons 18-27 | none identified | 93 |  |
| **UPEN-RB-246** | 25 | male | 3 | RB1^-/-^ | del of exons 1-27 | del of exons 1-27 | 92 |  |
| **UPEN-RB-242** | 8 | male | 3 | RB1^-/-^ | ex_10 g.64348C>T; c.958C>T (p.Arg320*) | ex_14 g.76430C>T c.1333C>T (p.Arg445*) | 91 |  |
| **UPEN-RB-221** | 25 | female | 3 | RB1^-/-^ | del exon 18 | LOH | 90 |  |
| **UPEN-RB-217** | 17 | female | 3 | RB1^-/-^ | ex_8 g.59701C>T; c.769C>T (p.Gln257*) | LOH | 89 |  |
| **UPEN-RB-197** | 24 | male | 3 | RB1^-/-^ | promoter methylation | LOH | 88 |  |
| **UPEN-RB-191** | 16 | male | 3 | RB1^-/-^ | del of exons 3-17 | del of exons 3-17 | 87 |  |
| **UPEN-RB-170** | 22 | female | 3 | RB1^-/-^ | ex_23 g.162248 C>G ; c.2370C>G (p.Tyr790*) | LOH | 86 |  |
| **UPEN-RB-144** | 43 | female | 3 | RB1^-/-^ | ex_13 g.73869G>T; c.1332G>T (p.Gln444His) | dup exons 1-27 | 85 |  |
| **UPEN-RB-123** | 9 | male | 3 | RB1^-/-^ | promoter methylation | LOH | 84 |  |
| **UPEN-RB-121** | 34 | female | 3 | RB1^-/-^ | ex_5 g.44675G>T; c.508G>T (p.Glu170*) | ex_14 g.76460C>T c.1363C>T (p.Arg455*) | 83 |  |
| **UPEN-RB-116** | 29 | female | 3 | RB1^-/-^ | ex_25 g.173711InsC; c.2525insC (p.Ser842Serfs*1) | LOH | 82 |  |
| **^1^UPEN-RB-08** | 5 | female | 3 | RB1^-/-^ | ex_20 g.156717T>C; c.1985T>C (p.Leu662Pro) | LOH | 81 |  |
| **UPEN-RB-91** | 26 | female | 3 | RB1^-/-^ | ex_15 g.76918delT; c.1419delT (p.Phe473Leufs*4) | LOH | 80 |  |
| **UPEN-RB-203** | 9 | female | 3 | RB1^-/-^ | ex_1 g.2172_2174delGCCinCT; c.113-115delGCCinsCT (p.Gly38Alafs*27) | LOH | 79 |  |
| **UPEN-RB-194** | 13 | female | 3 | RB1^-/-^ | ex_6 g.45844G>A; c.585G>A (p.Trp195*) | promoter methylation | 78 |  |
| **UPEN-RB-224** | 36 | female | 3 | RB1^-/-^ | in_6 g.45867G>A; c.607 + 1G>A | LOH | 77 |  |
| **UPEN-RB-222** | 4 | male | 3 | RB1^-/-^ | promoter methylation | LOH | 76 |  |
| **UPEN-RB-178** | 43 | male | 3 | RB1^-/-^ | in_19 g.156691A>T; c.1961 - 2A>T (skip exon 19) | LOH | 75 |  |
| **^1^UPEN-RB-36** | 23 | male | 3 | RB1^-/-^ | promoter methylation | LOH | 74 |  |
| **^1^UPEN-RB-29** | 2 | female | 3 | RB1^-/-^ | ex_11 g.65421T>C; c.1107T>C (p.Ile369Ile) | LOH | 73 |  |
| **^1^UPEN-RB-25** | 31 | female | 3 | RB1^-/-^ | ex_15 g.76898C>T; c.1399C>T (p.Arg467*) | ex_18 g.150025C>T c.1723C>T (p.Gln575*) | 72 |  |
| **^1^UPEN-RB-11** | 13 | male | 3 | RB1^-/-^ | ex_10 g.64348C>T; c.958C>T (p.Arg320*) | LOH | 71 |  |
| **UPEN-RB-57** | 24 | male | 3 | RB1^-/-^ | promoter methylation | LOH | 70 |  |
| **UPEN-RB-119** | 28 | male | 3 | RB1^+/-^ | in_24 g.170403G>A; c.2520 + 1G>A | none identified | 69 |  |
| **UPEN-RB-211** | 60 | female | 3 | RB1^-/-^ | in_13 g.73752G>A; c.1216 - 1G>A | ex_19 g.153352dupA c.1959dupA (p.Val654Serfs*14) | 68 |  |
| **UPEN-RB-209** | 32 | male | 3 | RB1^-/-^ | ex_15 g.76898C>T; c.1399C>T (p.Arg467*) | LOH | 67 |  |
| **UPEN-RB-195** | 18 | male | 3 | RB1^-/-^ | ex_3 g.39551_39552delAT; c.371-372del (p.Ile124Argfs*6) | LOH | 66 |  |
| **^1^UPEN-RB-46** | 19 | female | 3 | RB1^-/-^ | ex_4 g.41991C>G; c.446C>G (p.Ser149*) | LOH | 65 |  |
| **UPEN-RB-124** | 18 | female | 3 | RB1^-/-^ | in_22/ex_23 g.162203_162210del8; c.2326 - 1del-2332del8 | LOH | 64 |  |
| **^1^UPEN-RB-19** | 14 | male | 3 | RB1^-/-^ | ex_12 g.70264delC; c.1150delC (p.Gln384Asnfs*1) | LOH | 63 |  |
| **UPEN-RB-65** | 24 | female | 3 | RB1^-/-^ | ex_18 g.150037C>T; c.1735C>T (p.Arg579*) | LOH | 62 |  |
| **UPEN-RB-79** | 39 | female | 3 | RB1^+/-^ | ex_25 g.173713G>T; c2527G>T (p.Glu843*) | none identified | 61 |  |
| **UPEN-RB-129** | 21 | female | 3 | RB1^+/+^ | none identified | none identified | 60 |  |
| **UPEN-RB-156** | 26 | male | 3 | RB1^-/-^ | ex_17 g.78272G>A; c.1688G>A (p.Trp563*) | ex_19 g.153318T>A c.1925T>A (p.Leu642*) | 59 |  |
| **UPEN-RB-214** | 24 | female | 3 | RB1^-/-^ | ex_20 g.156705C>A; c.1973C>A (p.Ala658Asp) | LOH | 58 |  |
| **UPEN-RB-204** | 51 | female | 3 | RB1^-/-^ | ex_18 g.150037C>T; c.1735C>T (p.Arg579*) | LOH | 57 |  |
| **UPEN-RB-185** | 3 | female | 3 | RB1^-/-^ | ex_17 g.78081_78093del13; c.1499-2del_1509del13 (p.Arg500Lysfs*2) | LOH | 56 |  |
| **UPEN-RB-171** | 21 | female | 3 | RB1^-/-^ | del of exon 1 | del of exon 1 | 55 |  |
| **UPEN-RB-146** | 22 | female | 3 | RB1^-/-^ | ex_8 g.59695 C>T; c.763C>T (p.Arg255*) | LOH | 54 |  |
| **UPEN-RB-128** | 30 | male | 3 | RB1^-/-^ | promoter methylation | LOH | 53 |  |
| **^1^UPEN-RB-22** | 24 | male | 3 | RB1^-/-^ | ex_14 g.76460C>T; c.1363C>T (p.Arg455*) | LOH | 52 |  |
| **UPEN-RB-76** | 29 | male | 3 | RB1^-/-^ | promoter methylation | LOH | 51 |  |
| **UPEN-RB-137** | NA | female | 3 | RB1^+/-^ | promoter methylation | complex rearrangement | 50 |  |
| **UPEN-RB-248** | 6 | female | 3 | RB1^-/-^ | ex_18 g.150037C>T; c.1735C>T (p.Arg579*) | LOH | 49 |  |
| **UPEN-RB-226** | 108 | male | 3 | RB1^-/-^ | ex_18 g.150037C>T; c.1735C>T (p.Arg579*) | LOH | 48 |  |
| **UPEN-RB-219** | 35 | female | 3 | RB1^-/-^ | del exons 1-27 | promoter methylation | 47 |  |
| **UPEN-RB-184** | 31 | male | 3 | RB1^-/-^ | promoter methylation | LOH | 46 |  |
| **^1^UPEN-RB-32** | 13 | male | 3 | RB1^-/-^ | ex_4 g.41983_41986delTGCT c.438_441delTGCT (p.Asn146Lysfs*5) | ex_8 g.59695C>T; c.763C>T (p.Arg255*) | 45 |  |
| **^1^UPEN-RB-27** | 19 | male | 3 | RB1^-/-^ | ex_10 g.64348C>T; c.958C>T (p.Arg320*) | LOH | 44 |  |
| **UPEN-RB-112** | 6 | male | 3 | RB1^-/-^ | ex_8 g.59695C>T; c.763C>T (p.Arg255*) | LOH | 43 |  |
| **UPEN-RB-110** | 8 | male | 3 | RB1^-/-^ | ex_14 g.76460C>T; c.1363C>T (p.Arg455*) | LOH | 42 |  |
| **^1^UPEN-RB-14** | 66 | male | 3 | RB1^-/-^ | in_19 g.153358G>A; c.1960 + 5G>A | LOH | 41 |  |
| **UPEN-RB-94** | 58 | male | 3 | RB1^-/-^ | in_20 g.156839G>C; c.2106 + 1G>C | LOH | 40 |  |
| **UPEN-RB-63** | 24 | female | 3 | RB1^-/-^ | ex_1 g.2162C>T; c.103C>T (p.Gln35*) | LOH | 39 |  |
| **UPEN-RB-225** | 43 | male | 3 | RB1^+/-^ | ex_7 g.56866_56870 delAATGG; c.621-625delAATGG (p.Met208Argfs*3) | none identified | 38 |  |
| **UPEN-RB-240** | 10 | female | 3 | RB1^-/-^ | in_24 g.170403G>C; c.2520 + 1G>C | promoter methylation | 37 |  |
| **UPEN-RB-213** | 6 | male | 3 | RB1^-/-^ | promoter methylation | LOH | 36 |  |
| **UPEN-RB-207** | 17 | male | 3 | RB1^-/-^ | ex_12 g.70280T>A; c.1166T>A (p.Leu389*) | LOH | 35 |  |
| **UPEN-RB-143** | NA | female | 3 | RB1^-/-^ | ex_18 g.150037C>T; c.1735C>T (p.Arg579*) | LOH | 34 |  |
| **UPEN-RB-66** | 27 | female | 3 | RB1^+/-^ | in_18 g.150117G>A; c.1814 + 1G>A | none identified | 33 |  |
| **UPEN-RB-187** | 25 | male | 3 | RB1^+/+^ | none identified | none identified | 32 |  |
| **UPEN-RB-102** | 3 | male | 3 | RB1^-/-^ | ex_8 g.59683C>T; c.751C>T (p.Arg251*) | LOH | 31 |  |
| **UPEN-RB-68** | 12 | male | 3 | RB1^-/-^ | complex rearrangement | LOH | 30 |  |
| **UPEN-RB-59** | 11 | male | 3 | RB1^-/-^ | ex_2 g.5483_5484dupTA; c.197-198dupTA (p.Pro67Tyrfs*11) | ex_22 g.162012_162015 c.2227_2230dupTTGA (p.Leu743fs*9) | 29 |  |
| **UPEN-RB-52** | 4 | female | 3 | RB1^-/-^ | in_21 g.160839G>T; c.2211 + 5G>T | LOH | 28 |  |
| **UPEN-RB-134** | 31 | male | 3 | RB1^+/-^ | LOH | none identified | 27 |  |
| **UPEN-RB-188** | 40 | male | 2 | RB1^-/-^ | ex_10 g.64400dupT; c.1010dupT (p.Leu337Phefs*4) | ex_17 g.78144delG c.1560delG (p.Asn522Ilefs*2) | 26 |  |
| **UPEN-RB-120** | 6 | female | 2 | RB1^-/-^ | ex_21 g.160820C>T; c.2197C>T (p.His733Tyr) | ex_25 g.173712InsC c.2526insC (p.Glu843*) | 25 |  |
| **^1^UPEN-RB-30** | 12 | male | 2 | RB1^-/-^ | ex_8 g.59683C>T; c.751C>T (p.Arg251*) | LOH | 24 |  |
| **UPEN-RB-99** | 26 | male | 2 | RB1^-/-^ | ex_8 g.59791G>T; c.859G>T (p.Glu287*) | LOH | 23 |  |
| **UPEN-RB-237** | 16 | male | 2 | RB1^+/+^ | none identified | none identified | 22 |  |
| **^1^UPEN-RB-28** | 4 | male | 2 | RB1^-/-^ | ex_3 g.39486_39487delGT; c.305-306del (p.Cys102Tyrfs*7) | ex_19 g.153302C>T c.1909C>T (p.Gln637*) | 21 |  |
| **UPEN-RB-97** | 25 | male | 2 | RB1^-/-^ | promoter methylation | LOH | 20 |  |
| **^1^UPEN-RB-34** | 40 | male | 2 | RB1^-/-^ | ex_18 g.150002C>T; c.1700C>T (p.Ser567Leu) | LOH | 19 |  |
| **^1^UPEN-RB-24** | 33 | female | 2 | RB1^+/-^ | promoter methylation | none identified | 18 |  |
| **UPEN-RB-98** | NA | male | 2 | RB1^+/-^ | LOH | none identified | 17 |  |
| **UPEN-RB-106** | 37 | male | 2 | RB1^-/-^ | del ex 8,11,12,15,16, 20 | promoter methylation | 16 |  |
| **UPEN-RB-86** | 10 | female | 2 | RB1^-/-^ | ex_1 ~480 bases replacing exon 1 | ex_1 g.2090G>T; c.31G>T (p.Ala11Ser) | 15 |  |
| **UPEN-RB-108** | 18 | male | 2 | RB1^+/-^ | LOH | none identified | 14 |  |
| **^1^UPEN-RB-06** | 26 | male | 2 | RB1^+/-^ | ex_14 g.76460C>T; c.1363C>T (p.Arg455*) | none identified | 13 |  |
| **UPEN-RB-167** | 69 | female | 2 | RB1^+/+^ | none identified | none identified | 12 |  |
| **UPEN-RB-136** | 26 | female | 2 | RB1^-/-^ | promoter methylation | LOH | 11 |  |
| **UPEN-RB-174** | 31 | female | 2 | RB1^+/+^ | none identified | none identified | 10 |  |
| **UPEN-RB-233** | 7 | female | 2 | RB1^+/-^ | LOH | none identified | 9 |  |
| **UPEN-RB-107** | 20 | female | 2 | RB1^-/-^ | ex_17 g.78250C>T; c.1666C>T (p.Arg556*) | ex_18 g.150037C>T c.1735C>T (p.Arg579*) | 8 |  |
| **UPEN-RB-122** | 18 | female | 2 | RB1^-/-^ | in_13 g.73870G>T; c.1332 + 1G>T | ex_16 g.77051T>C c.1472T>C (p.Leu491Pro) | 7 |  |
| **UPEN-RB-218** | 7 | male | 2 | RB1^-/-^ | ex_10 g.64348C>T; c.958C>T (p.Arg320*) | promoter methylation | 6 |  |
| **UPEN-RB-220** | 0.5 | male | 2 | RB1^+/-^ | promoter methylation | none identified | 5 |  |
| **UPEN-RB-210** | 77 | male | 2 | RB1^+/-^ | complex rearrangement | none identified | 4 |  |
| **UPEN-RB-85** | 3 | male | 1 | RB1^-/-^ | in_19 g.153358G>A; c.1960 + 5G>A | LOH | 3 |  |
| **UPEN-RB-158** | 27 | male | 1 | RB1^-/-^ | ex_23 g.162237C>T; c.2359C>T (p.Arg787*) | LOH | 2 |  |
| **UPEN-RB-101** | 15 | male | 1 | RB1^+/+^ | none identified | none identified | 1 |  |
| ^1^ indicates tumors included in study by McEvoy J, Nagahawatte P, Finkelstein D, et al. *RB1* gene inactivation by chromothripsis in human retinoblastoma. Oncotarget. 2014; 2: 438-450. | | | | | | | | |

Supporting Table S2. Histopathological features of 111 retinoblastomas.

| **UPEN-RB_ID** | **Age at Diagnosis (months)** | ***MYCN* Copy Number** | **Number *RB*1 mutations** | **High Risk Features^a^** | **Growth Pattern^b^** | **Tumor Differentiation^c^** | **Rosettes^d^** | **Optic Nerve Invasion** | **Choroidal Invasion^e^** | **Anterior Chamber Involvement** | **Extra-Ocular or Extra-Scleral Extension** | **Other Invasion^f^** | **Tumor Seeding** |
| --- | --- | --- | --- | --- | --- | --- | --- | --- | --- | --- | --- | --- | --- |
| **UPEN-RB-125** | 1 | 128 | RB1+/+ |  | endo | PD | na | pre-laminar | na | na | na | na | na |
| **UPEN-RB-175** | 10 | 112 | RB1+/+ | yes | exo | mostly PD | focus HW | retrolaminar | massive | na | no | na | na |
| **UPEN-RB-127** | 13 | 108 | RB1+/+ |  | exo | PD | no | no | no | na | no | na | yes |
| **UPEN-RB-129** | 21 | 3 | RB1+/+ |  | na | undiff | no | no | no | no | no | no | na |
| **UPEN-RB-237** | 16 | 2 | RB1+/+ | yes | na | na | na | retrolaminar | na | na | na | na | na |
| **UPEN-RB-174** | 31 | 2 | RB1+/+ |  | na | UD | na | no | no | na | no | na | na |
| **UPEN-RB-93** | 5 | 101 | RB1+/- |  | exo | PD | no | no | no | na | no | na | na |
| **UPEN-RB-198** | 4 | 69 | RB1+/- |  | endo | PD | no | pre-laminar | no | na | no | na | yes |
| **UPEN-RB-40** | 9 | 59 | RB1+/- |  | combo | PD | no | no | non-massive | na | no | na | na |
| **UPEN-RB-115** | 24 | 30 | RB1+/- | yes | combo | PD | no | retrolaminar | non-massive | na | no | na | yes |
| **UPEN-RB-80** | 30 | 7 | RB1+/- |  | combo | mostly PD | no | no | no | na | na | na | na |
| **UPEN-RB-92** | 60 | 5 | RB1+/- | yes | endo | UD | na | no | non-massive | yes | no | iris | na |
| **UPEN-RB-61** | 19 | 4 | RB1+/- |  | na | PD | few FW | Pre-laminar | na | na | na | na | yes |
| **UPEN-RB-82** | 21 | 4 | RB1+/- |  | combo | mostly PD | few FW | no | no | na | no | na | na |
| **UPEN-RB-88** | 7 | 4 | RB1+/- |  | exo | mostly PD | focus FW | no | no | na | no | na | na |
| **UPEN-RB-180** | 22 | 4 | RB1+/- |  | combo | UD | na | pre-laminar | no | no | na | no | yes |
| **UPEN-RB-183** | 43 | 3 | RB1+/- |  | endo | PD | few FW | Pre-laminar | no | na | no | na | yes |
| **UPEN-RB-119** | 28 | 3 | RB1+/- |  | combo | WD | FW | no | no | no | na | na | yes |
| **UPEN-RB-79** | 39 | 3 | RB1+/- |  | exo | UD | na | lamina cribosa | no | na | no | na | na |
| **UPEN-RB-225** | 43 | 3 | RB1+/- |  | na | na | foci FW | no | non-massive | na | na | na | na |
| **UPEN-RB-134** | 31 | 3 | RB1+/- | yes | na | MD | na | lamina cribosa | no | yes | na | na | na |
| **UPEN-RB-06** | 26 | 2 | RB1+/- |  | na | na | FW | no | no | na | na | na | na |
| **UPEN-RB-233** | 7 | 2 | RB1+/- |  | exo | WD | na | no | no | no | no | no | na |
| **UPEN-RB-210** | 77 | 2 | RB1+/- | yes | na | na | na | na | massive | na | no | CB | na |
| **UPEN-RB-199** | 39 | 43 | RB1-/- | yes | diffuse infiltrating | PD | na | no | no | yes | no | iris, CB | yes |
| **UPEN-RB-05** | 25 | 42 | RB1-/- |  | combo | PD | no | post-laminar | no | na | no | na | na |
| **UPEN-RB-135** | 26 | 39 | RB1-/- | yes | combo | PD | no | no | no | yes | no | iris, CB | na |
| **UPEN-RB-153** | 40 | 35 | RB1-/- |  | combo | PD | na | no | non-massive | na | na | na | na |
| **UPEN-RB-201** | 9 | 30 | RB1-/- |  | combo | mixed | FW | pre-laminar | no | na | no | na | yes |
| **UPEN-RB-150** | 32 | 19 | RB1-/- |  | exo | MD | na | no | no | na | na | na | na |
| **UPEN-RB-159** | 28 | 15 | RB1-/- | yes | combo | PD | rare FW | pre-laminar | non-massive | no | no | na | na |
| **UPEN-RB-241** | 47 | 14 | RB1-/- | yes | indeterminate | indeterminate | na | retrolaminar | no | yes | no | iris, CB | na |
| **UPEN-RB-41** | 19 | 9 | RB1-/- |  | na | WD | FW | no | no | na | na | na | yes |
| **UPEN-RB-18** | 13 | 8 | RB1-/- |  | combo | mixed | some FW | no | no | na | no | na | na |
| **UPEN-RB-132** | 38 | 8 | RB1-/- | yes | combo | PD | no | retrolaminar | no | no | no | no | na |
| **UPEN-RB-84** | 37 | 7 | RB1-/- |  | exo | MD | FW | no | no | na | na | na | na |
| **UPEN-RB-104** | 60 | 7 | RB1-/- |  | combo | PD | no | no | no | na | no | na | yes |
| **UPEN-RB-133** | 24 | 6 | RB1-/- |  | combo | PD | no | no | no | na | no | na | yes |
| **UPEN-RB-148** | 42 | 6 | RB1-/- |  | endo | PD | no | pre-laminar | no | na | no | na | yes |
| **UPEN-RB-89** | 60 | 2 | RB1-/- | yes | exo | PD | few FW, HW | retrolaminar | massive | no | no | na | na |
| **UPEN-RB-75** | 27 | 5 | RB1-/- |  | endo | mostly PD | no | pre-laminar | no | na | na | na | yes |
| **UPEN-RB-169** | 76 | 5 | RB1-/- | yes | indeterminate | PD | few FW | retrolaminar | no | yes | no | iris | yes |
| **UPEN-RB-140** | 38 | 5 | RB1-/- |  | endo | PD | no | no | no | na | no | na | yes |
| **UPEN-RB-33** | 73 | 5 | RB1-/- |  | combo | PD | no | no | no | no | no | na | na |
| **UPEN-RB-83** | 11 | 5 | RB1-/- |  | endo | na | moderate FW | no | no | na | no | na | na |
| **UPEN-RB-04** | 17 | 5 | RB1-/- |  | exo | PD | few FW, HW | pre-laminar | no | na | no | na | na |
| **UPEN-RB-205** | 27 | 4 | RB1-/- |  | na | UD | na | no | no | na | no | na | yes |
| **UPEN-RB-70** | 28 | 4 | RB1-/- |  | endo | MD | few FW, HW | no | no | na | na | no | na |
| **UPEN-RB-105** | 48 | 4 | RB1-/- | yes | combo | PD | na | no | massive | no | yes | orbital | na |
| **UPEN-RB-166** | 32 | 4 | RB1-/- |  | exo | PD | no | no | no | no | no | na | na |
| **UPEN-RB-234** | 125 | 4 | RB1-/- | yes | combo | PD | na | retrolaminar | massive | na | no | na | yes |
| **UPEN-RB-71** | 30 | 4 | RB1-/- |  | exo | UD | no | no | no | na | no | na | no |
| **UPEN-RB-250** | 39 | 4 | RB1-/- |  | exo | PD | HW | pre-laminar | no | na | no | na | yes |
| **UPEN-RB-252** | 27 | 4 | RB1-/- |  | exo | PD | few HW | pre-laminar | non-massive | na | no | na | na |
| **UPEN-RB-72** | 24 | 4 | RB1-/- |  | endo | UD | no | no | no | na | na | na | na |
| **UPEN-RB-117** | 40 | 4 | RB1-/- | yes | combo | PD | na | retrolaminar | no | no | no | na | na |
| **UPEN-RB-74** | 54 | 4 | RB1-/- |  | combo | UD | na | no | no | na | na | na | na |
| **UPEN-RB-01** | 18 | 4 | RB1-/- | yes | exo | PD | na | retrolaminar | massive | no | no | na | na |
| **UPEN-RB-15** | 22 | 4 | RB1-/- |  | exo | mostly PD | no | no | no | na | no | na | na |
| **UPEN-RB-155** | 1 | 4 | RB1-/- |  | endo | WD | FW | pre-laminar | no | na | na | na | yes |
| **UPEN-RB-232** | 32 | 4 | RB1-/- |  | endo | PD | no | pre-laminar | no | na | na | na | yes |
| **UPEN-RB-251** | 27 | 4 | RB1-/- | yes | combo | mostly PD | rare | retrolaminar | no | na | no | na | yes |
| **UPEN-RB-39** | 34 | 4 | RB1-/- |  | endo | mixed | FW, HW | no | non-massive | na | no | na | na |
| **UPEN-RB-216** | 7 | 4 | RB1-/- | yes | combo | PD | no | retrolaminar | no | yes | no | na | na |
| **UPEN-RB-26** | 26 | 4 | RB1-/- |  | exo | mostly WD | na | no | no | na | na | na | na |
| **UPEN-RB-77** | 10 | 4 | RB1-/- |  | endo | mixed | FW, HW | no | no | na | no | na | na |
| **UPEN-RB-152** | 20 | 4 | RB1-/- |  | combo | PD | no | no | no | na | na | na | na |
| **UPEN-RB-190** | 24 | 4 | RB1-/- | yes | combo | PD | na | pre-laminar | non-massive | no | no | na | na |
| **UPEN-RB-149** | 30 | 4 | RB1-/- |  | exo | PD | few FW | no | no | na | na | na | na |
| **UPEN-RB-243** | 20 | 4 | RB1-/- |  | endo | PD | na | pre-laminar | no | na | no | na | yes |
| **UPEN-RB-69** | 9 | 4 | RB1-/- |  | exo | WD | FW | no | no | na | na | na | na |
| **UPEN-RB-103** | 8 | 4 | RB1-/- | yes | na | MD | FW, HW | retrolaminar | no | na | no | na | yes |
| **UPEN-RB-130** | 50 | 4 | RB1-/- | yes | combo | PD | HW | post-laminar | massive | no | no | CB | yes |
| **UPEN-RB-131** | 6 | 4 | RB1-/- |  | combo | PD | focus FW | no | no | na | no | na | na |
| **UPEN-RB-145** | 23 | 4 | RB1-/- |  | endo | na | na | pre-laminar | no | na | no | na | yes |
| **UPEN-RB-16** | 41 | 4 | RB1-/- |  | exo | PD | focus HW | no | no | na | no | na | na |
| **UPEN-RB-48** | 22 | 4 | RB1-/- |  | combo | PD | na | pre-laminar | no | no | no | no | na |
| **UPEN-RB-90** | 9 | 3 | RB1-/- |  | exo | MD | FW, HW | pre-laminar | no | no | no | no | no |
| **UPEN-RB-223** | 136 | 3 | RB1-/- | yes | combo | PD | scattered FW | retrolaminar | non-massive | na | no | no | yes |
| **UPEN-RB-60** | 3 | 3 | RB1-/- |  | combo | WD | FW | no | no | na | na | na | na |
| **UPEN-RB-09** | 19 | 3 | RB1-/- |  | na | PD | na | pre-laminar | no | na | na | na | na |
| **UPEN-RB-121** | 34 | 3 | RB1-/- |  | endo | WD | FW | pre-laminar | no | na | no | na | na |
| **UPEN-RB-144** | 43 | 3 | RB1-/- |  | endo | PD | few HW | lamina cribosa | no | na | no | na | yes |
| **UPEN-RB-217** | 17 | 3 | RB1-/- | yes | exo | mixed | FW | no | massive | na | no | na | yes |
| **UPEN-RB-246** | 25 | 3 | RB1-/- |  | combo | mostly PD | na | pre-laminar | no | na | no | na | yes |
| **UPEN-RB-91** | 26 | 3 | RB1-/- |  | endo | UD | na | no | no | na | no | na | na |
| **UPEN-RB-178** | 43 | 3 | RB1-/- |  | endo | PD | na | pre-laminar | no | na | no | na | yes |
| **UPEN-RB-29** | 2 | 3 | RB1-/- |  | exo | PD | scattered F-W | no | no | na | no | na | na |
| **UPEN-RB-57** | 24 | 3 | RB1-/- |  | combo | mostly PD | some HW | lamina cribosa | no | na | na | na | yes |
| **UPEN-RB-209** | 32 | 3 | RB1-/- |  | endo | PD | no | pre-laminar | no | na | no | na | yes |
| **UPEN-RB-46** | 19 | 3 | RB1-/- |  | combo | MD | FW, HW | no | non-massive | na | no | na | na |
| **UPEN-RB-128** | 30 | 3 | RB1-/- | yes | combo | mixed | na | retrolaminar | none | no | no | no | na |
| **UPEN-RB-156** | 26 | 3 | RB1-/- |  | endo | PD | no | pre-laminar | no | na | no | na | yes |
| **UPEN-RB-185** | 3 | 3 | RB1-/- |  | combo | WD | FW | pre-laminar | no | no | no | na | na |
| **UPEN-RB-76** | 29 | 3 | RB1-/- |  | endo | PD, focus of PRD | na | pre-laminar | no | na | na | na | yes |
| **UPEN-RB-219** | 35 | 3 | RB1-/- | yes | combo | PD | no | retrolaminar | no | na | no | na | na |
| **UPEN-RB-112** | 6 | 3 | RB1-/- |  | combo | WD | FW | no | no | na | na | na | na |
| **UPEN-RB-14** | 66 | 3 | RB1-/- |  | combo | PD | rare | no | no | na | no | na | na |
| **UPEN-RB-184** | 31 | 3 | RB1-/- |  | endo | mostly PD | na | lamina cribosa | no | no | no | no | yes |
| **UPEN-RB-226** | 108 | 3 | RB1-/- |  | diffuse | PD | HW | no | no | na | no | na | no |
| **UPEN-RB-27** | 19 | 3 | RB1-/- |  | endo | PD | no | lamina cribosa | no | na | na | na | na |
| **UPEN-RB-32** | 13 | 3 | RB1-/- |  | combo | WD | FW | pre-laminar | no | na | no | na | na |
| **UPEN-RB-94** | 58 | 3 | RB1-/- |  | combo | mixed with PRD | few FW | no | no | na | no | na | na |
| **UPEN-RB-207** | 17 | 3 | RB1-/- |  | na | mixed | na | no | na | na | na | na | yes |
| **UPEN-RB-120** | 6 | 2 | RB1-/- |  | na | undiff | foci HW | no | no | no | no | no | no |
| **UPEN-RB-28** | 4 | 2 | RB1-/- |  | endo | WD | FW | pre-laminar | no | no | no | na | na |
| **UPEN-RB-106** | 37 | 2 | RB1-/- | yes | na | na | HW | pre-laminar | non-massive | na | no | na | yes |
| **UPEN-RB-136** | 26 | 2 | RB1-/- |  | na | WD | na | no | no | no | no | na | na |
| **UPEN-RB-107** | 20 | 2 | RB1-/- |  | endo | PD | few HW | pre-laminar | no | no | no | na | yes |
| **UPEN-RB-122** | 18 | 2 | RB1-/- | yes | exo | mostly PD | few FW | pre-laminar | massive | no | na | na | na |
| **UPEN-RB-85** | 3 | 1 | RB1-/- |  | exo | WD | FW | no | no | na | no | no | na |

High risk features defined as invasion of the optic nerve (ON) to the level of the retrolamina, massive uveal/choroidal invasion and/or invasion of the anterior chamber (AC), iris or ciliary body (CB). (Kaliki S, Shields CL, Rojanaporn D, et al. High-Risk Retinoblastoma Based on International Classification of Retinoblastoma: Analysis of 519 Enucleated Eyes. Ophthalmology. 2013;120: 997-1003.)

Abbreviations: Exo, exophytic growth patterm; Endo endophytic growth pattern; Combo, combined Exo and Endo; WD, well differentiated tumor; PD, poorly differentiated tumor; MD, moderately differentiated; UD, undifferentiated; PRD, photoreceptor differentiation; CB, ciliary bodyFW, Flexner-Winterstein rosettes; HW, Homer-Wright rosettes; choroidal invasion: non-massive (<3mm diameter), massive (>3mm diameter)

Supporting Table S3. *RB1* gene mutations and high risk histological features in 18 tumors with *MYCN*-amplification.

| UPEN-RB-ID | Age at Diagnosis (Months) | *MYCN* Copy Number | *MYCN* Amplicon Size (MB) | *RB1* Mutations | *RB1* Mutation #1^1^ | *RB1* Mutation #2^1^ | Tumor Differentiation^2^ | High Risk Histopathological Features^3^ |
| --- | --- | --- | --- | --- | --- | --- | --- | --- |
| UPEN-RB-125 | 1 | 128 | NA | *RB1* (+/+) | none identified | none identified | PD | none |
| UPEN-RB -175 | 10 | 112 | NA | *RB1* (+/+) | none identified | none identified | mostly PD | Retrolaminar optic nerve and massive choroidal invasion |
| UPEN-RB-127 | 13 | 108 | NA | *RB1* (+/+) | none identified | none identified | PD | None |
| UPEN-RB-07 | 6 | 84 | 1.2 | *RB1* (+/+) | none identified | none identified | Pathology report NA | |
| UPEN-RB-176 | 7 | 65 | 2.4 | *RB1* (+/+) | none identified | none identified | Pathology report NA | |
| UPEN-RB-114 | 5 | 64 | 2.9 | *RB1* (+/+) | none identified | none identified | Pathology report NA | |
| UPEN-RB-198 | 4 | 69 | 1.6 | *RB1* (+/-) | rearrangement | none identified | PD | None |
| UPEN-RB-40 | 9 | 59 | 2.9 | *RB1* (+/-) | promoter methylation | none identified | PD | None |
| UPEN-RB-115 | 24 | 30 | 2.9 | *RB1* (+/-) | ex_8 p.Arg255* | none identified | PD | Retrolaminar optic nerve and non-massive choroidal invasion |
| UPEN-RB-93 | 5 | 101 | 2.1 | *RB1* (+/-) | LOH | none identified | PD | None |
| UPEN-RB-200 | 33 | 74 | NA | *RB1* (-/-) | ex_1 p.Ala14Ala | promoter methylation | Pathology report NA | |
| UPEN-RB-147 | 28 | 51 | 5.1 | *RB1* (-/-) | ex_3 p.Cys102Tyrfs*7 | in_13 c.1332+1G>T | Pathology report NA | |
| UPEN-RB-199 | 39 | 44 | 2 | *RB1* (-/-) | ex_17 p.Asp511Aspfs*7 | dup exon 1 | PD | Anterior chamber involvement; iris and ciliary body invasion |
| UPEN-RB-05 | 25 | 42 | 1.8 | *RB1* (-/-) | ex_15 p.Arg467* | LOH | PD | None |
| UPEN-RB-135 | 26 | 39 | 4.6 | *RB1* (-/-) | ex_4 p.Tyr155Leufs*2 | ex_17 p.Ser534SLysfs*21 | PD | Anterior chamber involvement; iris and ciliary body invasion |
| UPEN-RB-45 | 6 | 38 | 5 | *RB1* (-/-) | LOH | LOH | Pathology report NA | |
| UPEN-RB-153 | 40 | 35 | 1.5 | *RB1* (-/-) | ex_8 p.Arg255* | LOH | PD | None |
| UPEN-RB-201 | 9 | 30 | 11.1 | *RB1* (-/-) | ex_14 p.Arg455* | complex rearrangement | mixed | None |

^1^ LOH, loss of heterozygosity

^2^ PD, poorly differentiated tumor

^3^ High risk features, invasion of the optic nerve to the level of the retrolamina, massive uveal/choroidal invasion and/or invasion of the anterior chamber, iris or ciliary body. ). (Kaliki S, Shields CL, Rojanaporn D, et al. High-Risk Retinoblastoma Based on International Classification of Retinoblastoma: Analysis of 519 Enucleated Eyes. Ophthalmology. 2013;120: 997-1003.

Supporting Table 4. IHC staining of five retinoblastoma tumors with antibodies specific for SKP2 and p27.

| UPEN-RB-ID | *MYCN* Copy Number | *RB1* Mutations | Histo-pathological Features^1^ | High Risk Features | Immunohistochemical Staining Pattern of tumor | |
| --- | --- | --- | --- | --- | --- | --- |
|  |  |  |  |  | SKP2 | p27 |
| UPEN-RB-175 | 112 | *RB1*^+/+^ | PD-few HW rosettes; large cells with nucleoli, 50% necrotic | Invasion of optic nerve and choroid | very few moderately positive cells | diffuse weakly positive cells |
| UPEN-RB-93 | 101 | *RB1*^+/-^ | PD-no rosettes; cells with prominent nucleoli | None | very few moderately positive cells | patchy weakly positive cells |
| UPEN-RB-40 | 59 | *RB1*^+/-^ | PD-no rosettes; extensively necrotic | None | very few very weakly positive cells | about 40% moderately to strongly positive cells |
| UPEN-RB-201 | 30 | *RB1*^-/-^ | Mixed tumor differentiation; many FW rosettes | None | rosettes: negatve; PD tumor: scattered moderately positive | rosettes and PD tumor: strongly positive |
| UPEN-RB-112 | 3 | *RB1^-/-^* | WD with many FW rosettes; extensively | None | negative | patchy weakly positive cells |

^1^ Histopathological feature: WD, well differentiated tumor; PD, poorly differentiated tumor; FW, Flexner-Wintersteiner rosettes;

HW, Homer-Wright rosettes.
